# Near-isotropic polariton heat transport along a polar anisotropic nanofilm

**DOI:** 10.1016/j.isci.2022.104857

**Published:** 2022-08-05

**Authors:** Jose Ordonez-Miranda, Yunhui Wu, Masahiro Nomura, Sebastian Volz

**Affiliations:** 1 LIMMS, CNRS-IIS UMI 2820, The University of Tokyo, Tokyo 153-8505, Japan; 2 Institute of Industrial Science, The University of Tokyo, Tokyo 153-8505, Japan

**Keywords:** Heat transfer, Thermal property, Nanostructure

## Abstract

The heat transport of surface phonon-polaritons propagating along a polar uniaxial anisotropic nanofilm is studied for different orientations of its optical axis, film thicknesses, and temperatures. For an hBN nanofilm, it is shown that i) the propagation of polaritons can be described in terms of even and odd modes that generalize the transverse magnetic and transverse electrical ones that typically appear in isotropic films. ii) The frequency spectrum of polaritons can efficiently be tuned with the angle between the film optical axis and their propagation direction. iii) The polariton thermal conductivity takes higher values for a thinner or hotter nanofilm. iv) The even and odd modes have a remarkable contribution to the total polariton thermal conductivity, which takes a value higher than 5.6 Wm^−1^K^−1^ for a 25-nm-thick nanofilm at 500 K. The obtained results thus uncover some key features of the propagation and heat transport of polaritons in uniaxial nanofilms.

## Highlights

• Polariton thermal conductivity of a uniaxial anisotropic nanofilm
• Polariton heat transport driven by the optical axis of a polar anisotropic nanofilm
• Higher thermal conductivities for thinner or hotter nanofilms
• Near-isotropic thermal response of a strongly anisotropic nanofilm

## Graphical abstract

**Figure:**
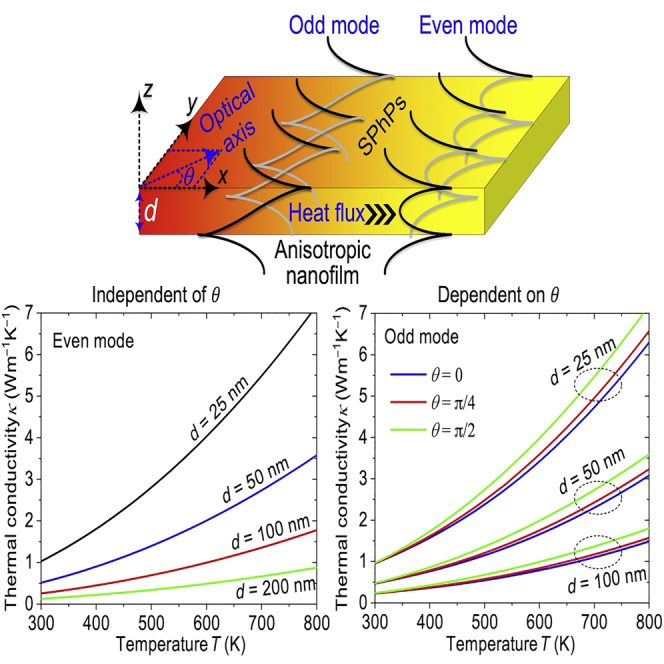

## Introduction

With the continuous development of electronic devices ever thinner, their operation under enhanced rates generates a significant overheating of the involved nanomaterials, which reduces their lifetime and increases the energy consumption through the use of cooling fans. This overheating is the result of the thermal performance reduction of the used materials as their sizes are scaled down to a few tens of nanometers (Volz et al., 2016a, 2016b). This problem of heat dissipation is a real industrial challenge that has boosted the study of nanoscale heat transfer and could partially be resolved by means of surface electromagnetic waves propagating along the interface of nanomaterials (Agranovich 2012; Chen et al., 2005; Liu et al., 2021). In polar nanomaterials (i.e. SiO_2_, SiC, SiN, and hexagonal boron nitride (hBN)), these evanescent waves are named surface phonon-polaritons (SPhPs) and are generated by the fluctuation of their microscopic electrical dipoles, which under a thermal excitation, oscillate and emit an electromagnetic field. This field induces the excitation of neighboring dipoles, which keep the propagation of the field along the material interfaces mainly (Ordonez-Miranda et al., 2014a, 2014b, 2014c; Ordonez-Miranda et al., 2013). In polar nanofilms, for instance, the increasing surface-to-volume ratio leads to a strong coupling of the SPhPs propagating along its both interfaces (Yang et al., 1991), which enlarges their propagation distance and therefore enhances their contribution to the in-plane heat transport (Chen et al., 2005; Greffet et al., 2002; Ordonez-Miranda et al., 2013). Recent experiments demonstrated that the SPhP thermal conductivity of suspended SiN and SiO_2_ nanofilms thinner than 50 nm can be comparable or even higher than their corresponding phonon counterparts (Tranchant et al., 2019; Wu et al., 2020).

Hyperbolic anisotropic media have recently attracted a lot of attention due to their ability to tailor the propagation of surface electromagnetic waves with the orientation of their optical axes (Jacob and Narimanov 2008; Li et al., 2008; Luo et al., 2013; Tao et al., 2021; Wang et al., 2011; Wu and Fu 2021; Zheng et al., 2020). Based on a sandwich structure made up of a graphene nanolayer deposited between two plates of hBN, Tao et al., (2021) showed that one can generate anisotropy-induced plasmon modes, whose propagation and polarization are driven by the angle between the optical axis (OA) of one of the uniaxial plates and the direction of propagation of the induced surface waves. The OA of a uniaxial crystal is one of its crystallographic axes in which the permittivity is different than that along the other two crystallographic axes. A uniaxial hBN film is thus isotropic within the plane orthogonal to its OA, whose direction can accurately be tuned to control the propagation features of polaritons (Luo et al., 2013; Ma et al., 2021; Wang et al., 2011). In hBN layers, the orientation of their OA was also used to strengthen the SPhP coupling (Barra-Burillo et al., 2021), control the energy flow in waveguides (Maia et al., 2019), and tune their optical response (Dai et al., 2014; Segura et al., 2018) in different directions. Biaxial anisotropic media, such as the van der Waals crystal *α*-MoO_3_, also allows us to improve the propagation and detection of surface electromagnetic waves (Ma et al., 2018; Zheng et al., 2020) through the orientation of their two optical axes and the expansion of the frequency window supporting their propagation to near- and mid-infrared frequencies (Zou et al., 2018). The orientation of the optical axes of uniaxial or biaxial anisotropic media thus represents a degree of freedom to tailor the propagation and energy transport of surface electromagnetic waves. As these parameters determine the thermal energy of SPhPs (Guo et al., 2021), the OA orientation is also expected to affect the SPhP thermal conductivity of anisotropic nanofilms; however, its impact is not explored yet.

In this work, we quantify the SPhP thermal conductivity of an hBN nanofilm for different orientations of its OA, thicknesses, and temperatures. This is done by deriving explicit expressions for the dispersion relation of SPhPs and finding analytical formulas for their propagation wavevector. It is shown that the OA orientation has a strong impact on the SPhP frequency spectrum, but a weak one on the overall values of the SPhP thermal conductivity. Higher conductivities are found for thinner and/or hotter nanofilms, which represents the fingerprint of the SPhP heat transport.

## Theoretical modeling

**Figure 1:**
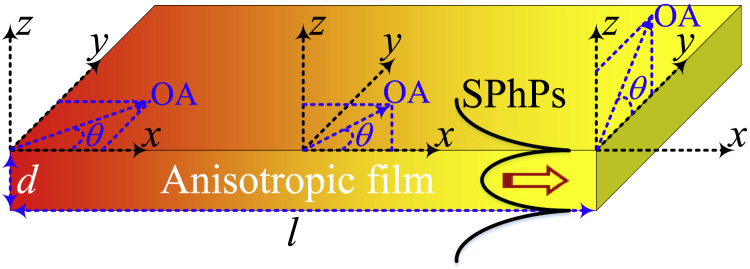
Scheme of a uniaxial anisotropic film supporting the propagation of SPhPs along its interfaces The film optical axis (OA) can be in the *xy*, *xz*, or *yz* plane.

Let us consider an anisotropic polar film of thickness *d* supporting the propagation of SPhPs along its interfaces, as shown in Figure 1. The film and its surrounding medium are non-magnetic (magnetic permeability equal to that of vacuum *μ*_0_), as is the case of air and hBN considered in this work (Caldwell et al., 2014). We consider that the SPhPs are thermally excited via the heating of the film surfaces *x* = 0 and l to activate the phonons supporting the existence and propagation of SPhPs in a broad range of frequencies (Tranchant et al., 2019). Assuming that these film surfaces are uniformly heated up, the heat propagates along the *x* axis mainly and the in-plane thermal conductivity of the film due to the SPhP propagation is given by (Guo et al., 2021)

(Equation 1) $$\kappa =\frac{1}{2\pi^{2}d}\int \hbar \omega \text{Re}\left(\beta \right)\Lambda_{e}\frac{\partial f}{\partial T}d\omega \text{,}$$

where ℏ is the Planck’s constant divided by 2*π*, Re(*β*) is the real part of the in-plane SPhP wavevector *β*, $f={\left[\exp\left(\hbar \omega /k_{B}T\right)-1\right]}^{-1}$ is the Bose-Einstein distribution function, *T* is the film average temperature, *k*_*B*_ is the Stefan-Boltzmann constant, *ω* is the spectral frequency, and $\Lambda_{e}$ is the effective propagation length determined by

(Equation 2) $$\Lambda_{e}=\frac{\pi }{2}\left(1-\frac{4\psi (0)}{\pi \lambda }\right)\Lambda ,$$

with $\lambda =l/\Lambda ,\;E_{n}\left(x\right)=\int_{0}^{\pi /2}\cos^{n-2}\left(\theta \right)e^{-x/\cos\left(\theta \right)}d\theta \text{.}$, being the SPhP intrinsic propagation length, $\psi \left(\xi \right)=E_{5}\left(\xi \right)-E_{5}\left(\lambda -\xi \right)\;\text{and}\;E_{n}\left(x\right)=\int_{0}^{\pi /2}\cos^{n-2}\left(\theta \right)e^{-x/\cos\left(\theta \right)}d\theta$. Equations (1) and (2) were derived under the assumption of one-dimensional heat transport imposed by external boundary conditions and therefore they are expected to be equally valid for isotropic and anisotropic films (Guo et al., 2021). Equation (2) thus establishes that the transmission of SPhPs along the film is driven by the ratio λ=l/Λ between its length *l* and Λ. In the diffusive regime $\left(\lambda \gg 1\right),\;\psi \left(0\right)/\lambda \to 0,\;\Lambda_{e}\approx \pi \Lambda /2,$ and Equation (2) reduce to the previous expression derived by Chen et al., (2005). In the ballistic limit (λ≪1), on the other hand, $1-4\psi \left(0\right)/\pi \lambda \approx 2\lambda /\pi \;\text{and}\;\Lambda_{e}\approx l\text{.}$ Therefore, the effective propagation length of SPhPs cannot exceed the film length *l*, even when their intrinsic propagation length Λ is much longer than *l*. The SPhP heat transport is hence enhanced along a film with long length *l* smaller than the SPhP propagation length (l≪Λ), as established by Equation (1). In general, according to Equations (1) and (2), the SPhP thermal conductivity depends on the material properties through the product $\text{Re}\left(\beta \right)\Lambda_{e}\left[2l\text{Im}\left(\beta \right)\right]$ determined by the SPhP wavevector I(I), which is the dispersion relation of SPhPs propagating along the film shown in Figure 1. As *κ* increases with this product, the optimal material configuration to maximize the SPhP heat transport is given by a large wavevector Re(*β*) and a long propagation length (small Im(I)). According to the Maxwell’s equations of electromagnetism, these two propagation parameters are expected to strongly depend on the orientation of the optical axis (OA) of the uniaxial anisotropic film and therefore we are going to consider its three cases shown in Figure 1.

Considering that there is no electrical source inside the film in Figure 1, the Maxwell’s equations describing the propagation of the SPhP electromagnetic fields take the form

(Equation 3a) $$\nabla \times \bar{E}+\frac{\partial \bar{B}}{\partial t}=0,$$

(Equation 3b) $$\nabla \times \bar{H}-\frac{\partial \bar{D}}{\partial t}=0,$$

(Equation 3c) $$\nabla \cdot \bar{D}=0,$$

(Equation 3d) $$\nabla \cdot \bar{B}=0,$$

where $\bar{B}=\mu_{0}\bar{H}\;\text{and}\;\bar{D}=\varepsilon_{0}\bar{\bar{\varepsilon }}\bar{E}$, with $\varepsilon_{0}$ being the permittivity of vacuum and $\bar{\bar{\varepsilon }}$ the film relative permittivity tensor defined by

(Equation 4) $$\bar{\bar{\varepsilon }}=\left[\begin{array}{lll} \varepsilon_{11} & \varepsilon_{12} & \varepsilon_{13}\\ \varepsilon_{21} & \varepsilon_{22} & \varepsilon_{23}\\ \varepsilon_{31} & \varepsilon_{32} & \varepsilon_{33} \end{array}\right].$$

Considering that the solutions of Equations (3a), (3b), (3c), and (3d) are electromagnetic waves with a wave vector $\bar{k}$, the fields can be decomposed as follows $\bar{F}\left(\bar{x},t\right)=\text{Re}\left[\bar{F}\exp\left(i\left(\bar{k}\cdot \bar{x}-\omega t\right)\right)\right]$. Under this decomposition, Equations (3a), (3b), (3c), and (3d) reduce to

(Equation 5a) $$\bar{k}\times \bar{E}-\omega \bar{B}=0,$$

(Equation 5b) $$\bar{k}\times \bar{H}+\omega \bar{D}=0,$$

(Equation 5c) $$\bar{k}\cdot \bar{D}=0,$$

(Equation 5d) $$\bar{k}\cdot \bar{B}=0.$$

Note that the scalar product of Equations (5a) and (5b) by $\bar{k}$ yields Equations (5c) and (5d), so from now on, we are going to consider Equations (5a) and (5b) only. The combination of these two latter equations yields the following expression for the electrical field amplitude $\bar{E}$

(Equation 6) $$\bar{k}\times \left(\bar{k}\times \bar{E}\right)+k_{0}^{2}\bar{\bar{\varepsilon }}\bar{E}=0,$$

where *k*_0_ = *ω*/*c* $k_{0}=\omega /c$ and $c=1/\sqrt{\varepsilon_{0}\mu_{0}}$ is the speed of light in vacuum. Taking into account that the SPhPs propagate along the x axis and must decay as they travel away from the film interfaces, their wave vector can be written as follows $\bar{k}=\left(\beta ,0,ip\right)$. The electrical field $\bar{E}\left(\bar{x},t\right)=\text{Re}\left[\bar{E}\exp\left(i\left(\beta x-\omega t\right)-pz\right)\right]$ thus exhibits its surface confinement through an exponential decay along the z axis, for Re(*p*) > 0. Under this condition, Equation (6) establishes that the components of the electrical field $\bar{E}=\left(E_{x},E_{y},E_{z}\right)$ are given by

(Equation 7) $$\left[\begin{array}{lll} p^{2}+\varepsilon_{11}k_{0}^{2} & \varepsilon_{12}k_{0}^{2} & i\beta p+\varepsilon_{13}k_{0}^{2}\\ \varepsilon_{21}k_{0}^{2} & p^{2}-p_{22}^{2} & \varepsilon_{23}k_{0}^{2}\\ i\beta p+\varepsilon_{31}k_{0}^{2} & \varepsilon_{32}k_{0}^{2} & -p_{33}^{2} \end{array}\right]\left[\begin{array}{l} E_{x}\\ E_{y}\\ E_{z} \end{array}\right]=0,$$

where $p_{nn}^{2}=\beta^{2}-\varepsilon_{nn}k_{0}^{2}$. As the non-trivial solution of Equation (7) $\left(M\bar{E}=0\right)$ is determined by the vanishing determinant of its 3 × 3 matrix *M*, the values of the transverse wavevector *p* are found from the condition |M|=0. After finding *p* and solving Equation (7) for two components of the electrical field in terms of its third one, the three components of the magnetic field $\bar{H}=\left(H_{x},H_{y},H_{z}\right)$ are determined by Equation (5a), which yields

(Equation 8) $$\mu_{0}\omega \left(H_{x},H_{y},H_{z}\right)=\left(-ipE_{y},ipE_{x}-\beta E_{z},\beta E_{y}\right).$$

The amplitude components of the electrical and magnetic fields can thus be expressed in terms of a single electrical field component that is determined by an external source. The specification of this source is, however, not required to derive the SPhP dispersion relation *β*(*ω*) established by the boundary conditions at the film interfaces z = ± *d*/2 (placing the origin of the z axis at the middle of the film). Considering that the spatial dependence of the electrical and magnetic fields along the z axis is respectively determined by $\tilde{E}=\bar{E}\exp\left(-pz\right)$ and $\tilde{H}=\bar{H}\exp\left(-pz\right)$, for each value of *p* (Agranovich 2012), these conditions are given by the continuity of the tangential field components, which read

(Equation 9a) $$z=-d/2:{\tilde{E}}_{x}={\tilde{E}}_{x}^{<},{\tilde{E}}_{y}={\tilde{E}}_{y}^{<},{\tilde{H}}_{x}={\tilde{H}}_{x}^{<},{\tilde{H}}_{y}={\tilde{H}}_{y}^{<},$$

(Equation 9b) $$z=d/2:{\tilde{E}}_{x}={\tilde{E}}_{x}^{>},{\tilde{E}}_{y}={\tilde{E}}_{y}^{>},{\tilde{H}}_{x}={\tilde{H}}_{x}^{>},{\tilde{H}}_{y}={\tilde{H}}_{y}^{>},$$

where the superscripts “<” and “>” stand for the fields below (z<−d/2) and above (z>d/2) the film. The proposed methodology will now be applied to find the fields and dispersion relations for each of the three OA orientations shown in Figure 1.

### OA in the *xy* plane

In this case, the OA is considered to be along *x*_0_ axis of the crystallographic coordinate system *x*_0_*y*_0_*z*_0_ of the uniaxial anisotropic film and therefore its relative permittivity tensor is this reference system can be written as follows

(Equation 10) $${\bar{\bar{\varepsilon }}}_{0}=\left[\begin{array}{lll} \varepsilon_{\|} & 0 & 0\\ 0 & \varepsilon_{⊥} & 0\\ 0 & 0 & \varepsilon_{⊥} \end{array}\right].$$

where $\varepsilon_{\|}$ and $\varepsilon_{⊥}$ are the permittivity components parallel and perpendicular to the OA. Taking into account that the SPhPs propagate along the x axis, which forms an angle *θ* with the *x*_0_ one, the permittivity tensor of the film in the SPhP coordinate system xyz is determined by a rotation around the z = z_0_ axis, as follows

(Equation 11) $$\bar{\bar{\varepsilon }}=R{\bar{\bar{\varepsilon }}}_{0}R^{-1}=\left[\begin{array}{lll} \varepsilon_{11} & \varepsilon_{12} & 0\\ \varepsilon_{21} & \varepsilon_{22} & 0\\ 0 & 0 & \varepsilon_{⊥} \end{array}\right].$$

where the rotating matrix *R* describing the transformation of coordinates in the *xy* plane is given by (Tao et al., 2021)

(Equation 12) $$R=\left[\begin{array}{lll} \cos\left(\theta \right) & -\sin\left(\theta \right) & 0\\ \sin\left(\theta \right) & \cos\left(\theta \right) & 0\\ 0 & 0 & 1 \end{array}\right].$$

After Equation (12) into Equation (11), one obtains the following nonzero permittivity components: $\varepsilon_{11}=\varepsilon_{\|}{\cos\;}^{2}\left(\theta \right)+\varepsilon_{⊥}{\sin\;}^{2}\left(\theta \right),\;\varepsilon_{22}=\varepsilon_{\|}{\sin\;}^{2}\left(\theta \right)+\varepsilon_{⊥}{\cos\;}^{2}\left(\theta \right),\;\text{and}\;\varepsilon_{12}=\varepsilon_{21}=\left(\varepsilon_{\|}-\varepsilon_{⊥}\right)\sin\left(\theta \right)\cos\left(\theta \right)\text{.}$ The angle *θ* between the SPhP propagation direction and the film OA thus represents a degree of freedom to tailor the SPhP propagation through a permittivity tensor with on- and off-diagonal elements. While *ε*_11_ and *ε*_22_ allow to combine the pro-polariton features of both $\varepsilon_{\|}$ and $\varepsilon_{⊥}$, $\varepsilon_{12}=\varepsilon_{21}$ inserts new characteristics that are fundamentally different from those of the non-rotated and isotropic cases, provided that θ≠0,π/2.

After reducing the M matrix in Equation (7) with the identifies *ε*_13_ = *ε*_23_ = *ε*_31_ = *ε*_32_ = 0, the condition |M|=0 yields

(Equation 13) $$\left(p^{2}-p_{22}^{2}\right)\left(p^{2}-\frac{\varepsilon_{11}}{\varepsilon_{33}}p_{33}^{2}\right)+\frac{\varepsilon_{12}\varepsilon_{21}}{\varepsilon_{33}}p_{33}^{2}k_{0}^{2}=0.$$

The transverse wavevector *p* has therefore four values that are strongly determined by the product *ε*_12_*ε*_21_ of the off-diagonal elements of the film permittivity tensor, such that

(Equation 14a) $$\varepsilon_{12}\varepsilon_{21}=0:p=\pm p_{22},p=\pm \sqrt{\frac{\varepsilon_{11}}{\varepsilon_{33}}}p_{33},$$

(Equation 14b) $$\varepsilon_{12}\varepsilon_{21}\neq 0:p=\pm \alpha_{+},\pm \alpha_{-},$$

where the roots *α*±are given by

(Equation 15a) $$\alpha_{\pm }=\sqrt{\chi \pm \sqrt{\chi^{2}-q}},$$

(Equation 15b) $$2\chi =p_{22}^{2}+\frac{\varepsilon_{11}}{\varepsilon_{33}}p_{33}^{2},$$

(Equation 15c) $$q=\frac{p_{33}^{2}}{\varepsilon_{33}}\left(\varepsilon_{11}p_{22}^{2}+\varepsilon_{12}\varepsilon_{21}k_{0}^{2}\right).$$

Note that, in the limit *ε*_12_*ε*_21_→0, the values of *α*± reduce to those of *p* in Equation (14a), as established by Equation (13).

#### *ε*_12_*ε*_21_ = 0

This condition appears for *θ* = 0,*π*/2 and allows splitting the field components in terms of transverse magnetic (TM) and transverse electrical (TE) waves, as shown below.

##### *p* = ± *p*_22_

In this case, Equation (7) $\left(M_{\pm }{\bar{E}}_{\pm }=0\right)$ written for each value of *p* = ± *p*_22_, establishes that $E_{x}^{\pm }=E_{z}^{\pm }=0$ and therefore $H_{y}^{\pm }=0$, as predicted by Equation (8). The only non-vanishing electrical field component *E*_*y*_ is thus perpendicular to the SPhP propagation direction (TE waves) and determines the magnetic field components *H*_*x*_ and *H*_*z*_ via Equation (8). As there are two values for *p*, the most general solutions for the tangential fields ${\tilde{E}}_{y}$ and ${\tilde{H}}_{x}$ are

(Equation 16a) $${\tilde{E}}_{y}=Ae^{-p_{22}z}+Be^{p_{22}z},$$

(Equation 16b) $$\mu_{0}\omega {\tilde{H}}_{y}=ip_{22}\left(-Ae^{-p_{22}z}+Be^{p_{22}z}\right),$$

where $A=E_{y}^{+}$, $B=E_{y}^{-}$, and the coefficients in Equation (16b) were derived by applying Equation (8). By analogy, the tangential components of the fields below (${\tilde{E}}_{y}^{<}$ ${\tilde{H}}_{x}^{<}$) and above (${\tilde{E}}_{y}^{>}$ ${\tilde{H}}_{x}^{>}$) the film can be written as follows

(Equation 17a) $${\tilde{E}}_{y}^{<}=A_{<}e^{p_{s}\left(z+d/2\right)}\text{,}$$

(Equation 17b) $$\mu_{0}\omega {\tilde{H}}_{y}^{<}=ip_{s}A_{<}e^{p_{s}\left(z+d/2\right)}\text{,}$$

(Equation 17c) $${\tilde{E}}_{y}^{>}=A_{>}e^{-p_{s}\left(z-d/2\right)}\text{,}$$

(Equation 17d) $$\mu_{0}\omega {\tilde{H}}_{y}^{>}=-ip_{s}A_{>}e^{-p_{s}\left(z-d/2\right)}\text{,}$$

where $p_{s}^{2}=\beta^{2}-\varepsilon_{s}k_{0}^{2}$, with $\varepsilon_{s}$ being the relative permittivity of the isotropic medium surrounding the film. The exponential functions in Equations (17a), (17b), (17c), and (17d) were chosen in such a way that the fields fulfill the physical constraint of decaying to zero for |z|→∞ and Re $\left(p_{s}\right)>0$. The combination of Equations (16) and (17) with the relevant boundary conditions in Equations (9a) and (9b) yields the following dispersion relations related to even $\left({\tilde{E}}_{y}\left(-z\right)={\tilde{E}}_{y}\left(z\right)\right)$ and odd $\left({\tilde{E}}_{y}\left(-z\right)=-{\tilde{E}}_{y}\left(z\right)\right)$ modes:

##### Even mode

According to the boundary conditions in (Equation 9a), (Equation 9b) and the fields in (Equation 16a), (Equation 16b) and (17), this symmetric mode is determined by ${\tilde{E}}_{y}\left(z\right)=2A\text{cosh}\left(p_{22}z\right)$ and the dispersion relation

(Equation 18) $$p_{s}+p_{22}\text{tanh}\left(p_{22}d/2\right)=0.$$

The SPhP dispersion relation of this mode of the TE waves thus depends on the film permittivity via its *ε*_22_ component only. By writing the in-plane wavevector as $\beta =k_{0}\sqrt{\varepsilon }$, the transverse wavevectors take the form $p_{l}=k_{0}\sqrt{\varepsilon -\varepsilon_{l}}$ and Equation (18) becomes

(Equation 19) $$\sqrt{\varepsilon -\varepsilon_{s}}+\sqrt{\varepsilon -\varepsilon_{22}}\text{tanh}\left(\sqrt{\varepsilon -\varepsilon_{22}}\lambda \right)=0\text{,}$$

where $\lambda =k_{0}d/2$ is the normalized film thickness. Equation (19) can analytically be solved by means of the perturbation method for λ < 1, which is the case of interest in this work, to strength the coupling of SPhPs propagating along the film interfaces and therefore to enhance their contribution to the film thermal conductivity defined in Equation (1). In practice, this condition (λ < 1) is usually well satisfied by polar nanofilms thinner than 100 nm (Ordonez-Miranda et al. 2013, 2014b). For an approximation up to λ_4_, Equation (19) takes the form

(Equation 20) $$\sqrt{\varepsilon -\varepsilon_{s}}+\left(\varepsilon -\varepsilon_{22}\right)\lambda \left(1-\frac{\varepsilon -\varepsilon_{22}}{3}\lambda^{2}\right)=0$$

Equation (20) thus indicates that the effective permittivity *ε* is given by the following series expansion

(Equation 21) $$\varepsilon =\varepsilon_{s}+\varepsilon^{\left(2\right)}\lambda^{2}+\varepsilon^{\left(4\right)}\lambda^{4}.$$

By inserting Equation (21) into Equation (20), one finds

(Equation 22a) $$\varepsilon^{\left(2\right)}={\left(\varepsilon_{22}-\varepsilon_{s}\right)}^{2},$$

(Equation 22b) $$\varepsilon^{\left(4\right)}=-\frac{4}{3}{\left(\varepsilon_{22}-\varepsilon_{s}\right)}^{3}.$$

Equations (21) and (22) establish that the leading terms of the transverse wavevectors are $p_{s}\to k_{0}\left(\varepsilon_{22}-\varepsilon_{s}\right)\lambda$, $p_{22}\to k_{0}\sqrt{\varepsilon_{s}-\varepsilon_{22}}$, which explicitly indicate that, in a lossless film (Im (*ε*_22_) = 0), this even mode does not support the surface confinement (Re($p_{s}$) $=p_{s}>0$ and Re(*p*_22_) =*p*_22_>0 ) of the TE waves and therefore there are no SPhPs. On the other hand, for a lossy film (Im(*ε*_22_) > ), as is the case of hBN considered in this work, these TE waves do support the propagation of SPhPs for frequencies fulfilling the condition $\text{Re}\left(\varepsilon_{22}\right)>\varepsilon_{s}$ (Re(*p*_*s*_) >0). This condition usually appears at frequencies much lower and higher than the resonance one of $\text{Re}\left(\varepsilon_{22}\right)$, for which $\text{Im}\left(\varepsilon_{22}\right)\to 0$. In this case, $\text{Re}\left(p_{22}\right)\to 0.5k_{0}\text{Im}\left(\varepsilon_{22}\right)/\sqrt{\text{Re}\left(\varepsilon_{22}\right)-\varepsilon_{s}}>0$, which confirms that these TE waves are actually SPhPs with weak surface confinement.

##### Odd mode

According to the boundary conditions in (Equation 9a), (Equation 9b) and the fields in (Equation 16a), (Equation 16b) and (Equation 17a), (Equation 17b), (Equation 17c), (Equation 17d), this antisymmetric mode is defined by ${\tilde{E}}_{y}\left(z\right)=2B\text{sinh}\left(p_{22}z\right)$ and the dispersion relation

(Equation 23) $$p_{22}+p_{s}\text{tanh}\left(p_{22}d/2\right)=0.$$

By applying the perturbation method to Equation (23), as we did it for the even mode in Equation (18), one can show that the odd mode of the TE waves does not have a solution for λ<1 and therefore it does not support the propagation of SPhPs along nanofilms. This mode hence does not contribute to the SPhP thermal conductivity of the film.

##### $p=\pm \sqrt{\varepsilon_{11}/\varepsilon_{33}p_{33}}\equiv \pm q_{22}$

In this case, Equation (7) $\left(M_{\pm }{\bar{E}}_{\pm }=0\right)$ determines that $E_{y}^{\pm }=0\;\text{and}\;E_{z}^{\pm }=\pm \sqrt{\varepsilon_{11}/\varepsilon_{33}}\beta E_{x}^{\pm }/p_{33}\text{,}$ for which Equation (8) establishes that $H_{x}^{\pm }=H_{z}^{\pm }=0$ and $\mu_{0}\omega H_{y}^{\pm }=\pm i\sqrt{\varepsilon_{11}\varepsilon_{33}}k_{0}^{2}E_{x}^{\pm }/p_{33}$. The only non-vanishing magnetic field component *H*_*y*_ is thus perpendicular to the SPhP propagation direction (TM waves). For the two values of *p*, the most general solutions for the tangential fields ${\tilde{E}}_{x}$ and ${\tilde{H}}_{y}$ are thus given by

(Equation 24a) $${\tilde{E}}_{x}=Ae^{-q_{22}z}+Be^{q_{22}z},$$

(Equation 24b) $$\mu_{0}\omega p_{33}{\tilde{H}}_{y}=i\sqrt{\varepsilon_{11}\varepsilon_{33}}k_{0}^{2}\left(Ae^{-q_{22}z}-Be^{q_{22}z}\right).$$

Similarly, the components of the tangential fields below (${\tilde{E}}_{x}^{<}$ ${\tilde{H}}_{y}^{<}$) and above (${\tilde{E}}_{x}^{>}$ ${\tilde{H}}_{y}^{>}$) the film can be written as follows

(Equation 25a) $${\tilde{E}}_{x}^{<}=A_{<}e^{p_{s}\left(z+d/2\right)}\text{,}$$

(Equation 25b) $$\mu_{0}\omega p_{s}{\tilde{H}}_{y}^{<}=-i\varepsilon_{s}k_{0}^{2}A_{<}e^{p_{s}\left(z+d/2\right)}\text{,}$$

(Equation 25c) $${\tilde{E}}_{x}^{>}=A_{>}e^{-p_{s}\left(z-d/2\right)}\text{,}$$

(Equation 25d) $$\mu_{0}\omega p_{s}{\tilde{H}}_{y}^{>}=i\varepsilon_{s}k_{0}^{2}A_{>}e^{-p_{s}\left(z-d/2\right)}\text{,}$$

where the transverse wavevector $p_{s}$ in the surrounding medium is defined just underneath Equation (17d). The combination of Equations (24) and (25) with the boundary conditions in Equations (9a) and (9b) yields the following dispersion relations related to even $\left({\tilde{H}}_{y}\left(-z\right)={\tilde{H}}_{y}\left(z\right)\right)$ and odd $\left({\tilde{H}}_{y}\left(-z\right)=-{\tilde{H}}_{y}\left(z\right)\right)$ modes:

##### Even mode

According to the boundary conditions in (Equation 9a), (Equation 9b) and the fields in Equations (24) and (25), the dispersion relation of this symmetric mode $\left({\tilde{H}}_{y}\left(-z\right)={\tilde{H}}_{y}\left(z\right)\right)$ is

(Equation 26) $$\sqrt{\varepsilon_{11}\varepsilon_{33}}p_{s}+\varepsilon_{s}p_{33}\text{tanh}\left(\sqrt{\varepsilon_{11}/\varepsilon_{33}}p_{33}d/2\right)=0.$$

In contrast to the even mode of the TE waves (see Equation (18)), the dispersion relation of this even mode does depends on two film permittivity components *ε*_11_ and *ε*_33_. This anisotropy has, however, a weak impact of wavevector $\beta =k_{0}\sqrt{\varepsilon }$ of SPhPs propagating along a thin enough nanofilm $\left(\lambda =k_{0}d/2<1\right)$, as established by the perturbation method. By expanding the hyperbolic tangent in Equation (26) in a power series of *λ* and following a similar procedure to the one developed in subsection p = ± p22 above, this method yields (for an approximation up to *λ*_4_)

(Equation 27) $$\varepsilon =\varepsilon_{s}+\varepsilon_{s}^{2}{\left(1-\frac{\varepsilon_{s}}{\varepsilon_{33}}\right)}^{2}\lambda^{2}+2\varepsilon_{s}^{2}{\left(1-\frac{\varepsilon_{s}}{\varepsilon_{33}}\right)}^{3}\left(\frac{\varepsilon_{11}}{3}-\frac{\varepsilon_{s}^{2}}{\varepsilon_{33}}\right)\lambda^{4}.$$

Equation (27) thus explicitly shows that the SPhP propagation is driven by the permittivity component *ε*_33_ mainly, as the nanofilm thickness scales down. The confinement condition $\text{Re}\left(p_{s}\right)>0$ with $p_{s}=k_{0}\sqrt{\varepsilon -\varepsilon_{s}}$ establishes that the TM waves of this even mode are SPhPs for all frequencies satisfying the condition ${\left|\varepsilon_{33}\right|}^{2}-\varepsilon_{s}\text{Re}\left(\varepsilon_{33}\right)>0$, which also holds for isotropic nanofilms (Ordonez-Miranda et al., 2021).

##### Odd mode

According to the boundary conditions in (Equation 9a), (Equation 9b) and the fields in Equations (24) and (25), this antisymmetric mode $\left({\tilde{H}}_{y}\left(-z\right)=-{\tilde{H}}_{y}\left(z\right)\right)$ is defined by the following dispersion relation

(Equation 28) $$\varepsilon_{s}p_{33}+\sqrt{\varepsilon_{11}\varepsilon_{33}}p_{s}\text{tanh}\left(\sqrt{\varepsilon_{11}/\varepsilon_{33}}p_{33}d/2\right)=0.$$

By applying the perturbation method, one finds that Equation (28) does not have a solution for the SPhP wavevector $\beta =k_{0}\sqrt{\varepsilon }$, as is the case of the odd mode of the TE waves. The odd mode of the TM waves is thus not of interest for determining the SPhP thermal conductivity considered in this work.

#### *ε*_12_*ε*_21≠0_

In this case, Equation (7) establishes that the amplitude components of the electrical field are related by $p_{33}^{2}E_{z\pm }^{\pm }=\pm i\beta \alpha_{\pm }E_{x\pm }^{\pm }$ and $\varepsilon_{21}k_{0}^{2}E_{x\pm }^{\pm }=\left(p_{22}^{2}-\alpha_{\pm }^{2}\right)E_{y\pm }^{\pm }$, where $\left(E_{x+}^{\pm },E_{y+}^{\pm },E_{z+}^{\pm }\right)$ and $\left(E_{x-}^{\pm },E_{y-}^{\pm },E_{z-}^{\pm }\right)$ are defined for *p* = ± *α*_+_ and *p* = ± *α*_−_, respectively. In contrast to the TE and TM waves considered in subsections p = ± p22 and p=±ε11/ε33p33≡±q22 above, here any of the electrical field components vanishes and therefore the SPhP propagation can no longer be described in terms of these waves, for any of the four roots of *p*. The tangential components of the electrical field below $\left({\tilde{E}}_{x}^{<},{\tilde{E}}_{y}^{<}\right)$, inside $\left({\tilde{E}}_{x},{\tilde{E}}_{y}\right)$, and above $\left({\tilde{E}}_{x}^{>},{\tilde{E}}_{y}^{>}\right)$ the film can therefore be written as follows

(Equation 29a) $${\tilde{E}}_{y}=A_{+}e^{-\alpha_{+}z}+A_{-}e^{\alpha_{+}z}+B_{+}e^{-\alpha_{-}z}+B_{-}e^{\alpha_{-}z},$$

(Equation 29b) $$\begin{array}{l} \varepsilon_{21}k_{0}^{2}{\tilde{E}}_{x}=\left(p_{22}^{2}-\alpha_{+}^{2}\right)\left(A_{+}e^{-\alpha_{+}z}+A_{-}e^{\alpha_{+}z}\right)+\\ \left(p_{22}^{2}-\alpha_{-}^{2}\right)\left(B_{+}e^{-\alpha_{-}z}+B_{-}e^{\alpha_{-}z}\right), \end{array}$$

(Equation 29c) $$\frac{{\tilde{E}}_{x}^{<}}{A_{<}}=\frac{{\tilde{E}}_{y}^{<}}{B_{<}}=e^{p_{s}\left(z+d/2\right)},$$

(Equation 29d) $$\frac{{\tilde{E}}_{x}^{>}}{A_{>}}=\frac{{\tilde{E}}_{y}^{>}}{B_{>}}=e^{-p_{s}\left(z-d/2\right)}.$$

In addition, the corresponding tangential components of the magnetic field can readily be obtained by means of Equations (8) and (29), and hence its explicit expressions will be omitted here, for the sake of conciseness. After inserting the tangential components of the electrical and magnetic fields into the boundary conditions in Equations (9a) and (9b), one obtains a system of eight equations, whose solutions yields the following dispersion relations

(Equation 30a) $$\begin{array}{l} \left(p_{22}^{2}-\alpha_{+}^{2}\right)\left(\frac{\varepsilon_{33}p_{s}\alpha_{+}+\varepsilon_{s}p_{33}^{2}\text{tanh}(\alpha_{+}d/2)}{\alpha_{+}+p_{s}\text{tanh}(\alpha_{+}d/2)}\right)=\\ \left(p_{22}^{2}-\alpha_{-}^{2}\right)\left(\frac{\varepsilon_{33}p_{s}\alpha_{-}+\varepsilon_{s}p_{33}^{2}\text{tanh}(\alpha_{-}d/2)}{\alpha_{-}+p_{s}\text{tanh}(\alpha_{-}d/2)}\right), \end{array}$$

(Equation 30b) $$\begin{array}{l} \left(p_{22}^{2}-\alpha_{+}^{2}\right)\left(\frac{\varepsilon_{s}p_{33}^{2}+\varepsilon_{33}p_{s}\alpha_{+}\text{tanh}(\alpha_{+}d/2)}{p_{s}+\alpha_{+}\text{tanh}(\alpha_{+}d/2)}\right)=\\ \left(p_{22}^{2}-\alpha_{-}^{2}\right)\left(\frac{\varepsilon_{s}p_{33}^{2}+\varepsilon_{33}p_{s}\alpha_{-}\text{tanh}(\alpha_{-}d/2)}{p_{s}+\alpha_{-}\text{tanh}(\alpha_{-}d/2)}\right). \end{array}$$

These transcendental equations for the SPhP wavevector *β*(*ω*) cannot be solved analytically for an arbitrary film thickness *d*, due to their hyperbolic tangents. However, for a thin nanofilm $\left(\lambda =k_{0}d/2<1\right)$ of interest in this work, the Taylor series expansion of these tangents in power of *λ* enables to find explicit expressions for *β* by applying the perturbation method used in subsection . For an approximation up to *λ*_4_, this method predicts that the effective permittivity $\varepsilon ={\left(\beta /k_{0}\right)}^{2}$ of the SPhP mode in Equation (30a) is precisely given by Equation (27) derived for the even mode of the TM waves found for *θ* = 0;*π*/2. As the leading coefficient of *λ*^2^ in Equation (27) depends on the film permittivity through $\varepsilon_{33}=\varepsilon_{⊥}$ only, this fact indicates that the SPhP mode in Equation (30a) is pretty much independent of the OA orientation for a thin enough nanofilm. On the other hand, the perturbation solution of Equation (30b) in terms of the effective permittivity *ε* is given by Equations (21) and (Equation 22a), (Equation 22b) that were derived for the even mode of the TE waves. Therefore, in contrast to the SPhP mode in Equation (30a), the one in Equation (30b) does depend on the OA orientation, given that the leading coefficient of *λ*^2^ in Equation (21) is driven by the film permittivity component *ε*_22_(*θ*) that depends on the angle *θ*, as established just below Equation (12). The SPhP modes in Equations (30a) and (30b) are thus the respective generalization of the TM and TE modes that show up when the SPhP coordinate axes (*x*,*y*,*z*) are aligned with the crystallographic ones of the anisotropic film (*θ* = 0;*π*/2). This is reasonable, given that the roots of *p* in Equation (13) reduce to those found for the TM and TE waves, in the limit *ε*_12_*ε*_21_→0. Furthermore, the fact that, for each mode, the leading term of the SPhP wavevector *β* depends on a single component of the film permittivity tensor indicates that the anisotropic effects are only weakly present on the SPhP propagation along a thin enough nanofilm.

### OA in the xz plane

In this case, the permittivity tensor of the film in the SPhP coordinate system xyz is determined by a rotation around the *y* = *y*_0_ axis (see Figure 1) and therefore it is given by

(Equation 31) $$\bar{\bar{\varepsilon }}=R{\bar{\bar{\varepsilon }}}_{0}R^{-1}=\left[\begin{array}{lll} \varepsilon_{11} & 0 & \varepsilon_{13}\\ 0 & \varepsilon_{⊥} & 0\\ \varepsilon_{31} & 0 & \varepsilon_{33} \end{array}\right].$$

where the rotating matrix *R* describing the transformation of coordinates in the *xz* plane is given by (Tao et al., 2021)

(Equation 32) $$R=\left[\begin{array}{lll} \cos\left(\theta \right) & 0 & -\sin\left(\theta \right)\\ 0 & 1 & 0\\ \sin\left(\theta \right) & 0 & \cos\left(\theta \right) \end{array}\right].$$

The combination of Equations (10), (31) and (32) yields the following nonzero permittivity components: $\varepsilon_{11}=\varepsilon_{\|}{\cos\;}^{2}\left(\theta \right)+\varepsilon_{⊥}{\sin\;}^{2}\left(\theta \right)$, $\varepsilon_{33}=\varepsilon_{\|}{\sin\;}^{2}\left(\theta \right)+\varepsilon_{⊥}{\cos\;}^{2}\left(\theta \right)$, and $\varepsilon_{13}=\varepsilon_{31}=\left(\varepsilon_{\|}-\varepsilon_{⊥}\right)\sin\left(\theta \right)\cos\left(\theta \right)$. As in the case of the OA in the xy plane, the off-diagonal elements $\varepsilon_{13}=\varepsilon_{31}$ vanish for *θ* = 0,*π*/2, that is to say, when the film crystallographic axes are parallel to the SPhP coordinate axes x,y,z. After reducing the *M* matrix in Equation (7) with the identifies $\varepsilon_{12}=\varepsilon_{21}=\varepsilon_{23}=\varepsilon_{32}=0$, the condition |M|=0 yields

(Equation 33a) $$p=\pm p_{22},$$

(Equation 33b) $$p=p_{\pm }=i\chi \pm \sqrt{q-\chi^{2}},$$

where the parameters *χ* and *q* are defined by

(Equation 34a) $$2\chi =\beta \frac{\varepsilon_{13}+\varepsilon_{31}}{\varepsilon_{33}},$$

(Equation 34b) $$q=\frac{\varepsilon_{11}p_{33}^{2}+\varepsilon_{13}\varepsilon_{31}k_{0}^{2}}{\varepsilon_{33}}.$$

Note that the two roots in Equation (33a) correspond to the ones driving the propagation of TE waves considered in Equations (14a) and (14b), while those in Equation (33b) reduce to $p=\pm \sqrt{\varepsilon_{11}/\varepsilon_{33}p_{33}}$, for $\varepsilon_{13}$ and $\varepsilon_{31}\to 0$. This fact indicates that the SPhP propagation for *θ* = 0,*π*/2 can be described in terms of TE and TM waves, as established by Equation (14a) and the results obtained in Equations (14a) and (14b). For 0≤θ≤π/2, on the other hand, Equation (33a) establishes that two of the SPhP dispersion relations are still given by the even and odd modes of the TE waves in Equations (18) and (23), provided that the permittivity component $\varepsilon_{22}=\varepsilon_{⊥}$, as defined by Equation (31). After solving Equation (7) for the electrical field components related to the other two roots of *p* in Equation (33b) and applying the boundary conditions in Equations (9a) and (9b) for the relevant tangential field components, as we did it in subsection ε12ε21 = 0, one obtains the following additional dispersion relation

(Equation 35) $$\begin{array}{ll} & \frac{p_{s}\left(\varepsilon_{33}p_{+}-i\varepsilon_{31}\beta \right)+\varepsilon_{s}p_{33}^{2}\text{tanh}\left(p_{+}d/2\right)}{\varepsilon_{s}p_{33}^{2}+p_{s}\left(\varepsilon_{33}p_{+}-i\varepsilon_{31}\beta \right)\text{tanh}\left(p_{+}d/2\right)}=\\ & \frac{p_{s}\left(\varepsilon_{33}p_{-}-i\varepsilon_{31}\beta \right)+\varepsilon_{s}p_{33}^{2}\text{tanh}\left(p_{-}d/2\right)}{\varepsilon_{s}p_{33}^{2}+p_{s}\left(\varepsilon_{33}p_{-}-i\varepsilon_{31}\beta \right)\text{tanh}\left(p_{-}d/2\right)}. \end{array}$$

In the limit of $\varepsilon_{13}=\varepsilon_{31}=0$, Equation (35) splits into the dispersion relations of the TE and TM waves defined respectively in Equations (26) and (28), as expected. These asymptotic dispersion relations of the TE and TM waves are the corresponding ones of the ordinary and extraordinary modes reported in the literature for a uniaxil film (Alvarez-Perez et al., 2019). In addition, the application of the perturbation method to Equation (35) indicates that SPhP propagation along a thin nanofilm $\left(\lambda =k_{0}d/2<1\right)$ is driven by the effective permittivity *ε* = (*β*/*k*_0_)^2^ given in Equation (27). This fact confirms that, as in the case of the OA in the xy plane, the propagation and therefore the energy transport of SPhPs along a thin enough nanofilm can be described in terms of even (TM-ike) and odd (TE-like) waves, regardless of the orientation of its OA.

### OA in the yz plane

Considering that the OA of the film is along the *y*_0_ axis of the crystallographic coordinate system *x*_0_*y*_0_*z*_0_, its relative permittivity tensor in this reference system can be written as follows

(Equation 36) $${\bar{\bar{\varepsilon }}}_{0}=\left[\begin{array}{lll} \varepsilon_{⊥} & 0 & 0\\ 0 & \varepsilon_{\|} & 0\\ 0 & 0 & \varepsilon_{⊥} \end{array}\right].$$

Therefore, according to Figure 1, the permittivity tensor in the SPhP coordinate system xyz is determined by a rotation around the *x* = *x*_0_ axis and is given by

(Equation 37) $$\bar{\bar{\varepsilon }}=R{\bar{\bar{\varepsilon }}}_{0}R^{-1}=\left[\begin{array}{lll} \varepsilon_{⊥} & 0 & 0\\ 0 & \varepsilon_{22} & \varepsilon_{23}\\ 0 & \varepsilon_{32} & \varepsilon_{33} \end{array}\right].$$

where the rotating matrix *R* describing the transformation of coordinates in the *yz* plane reads

(Equation 38) $$R=\left[\begin{array}{lll} 1 & 0 & 0\\ 0 & \cos\left(\theta \right) & -\sin\left(\theta \right)\\ 0 & \sin\left(\theta \right) & \cos\left(\theta \right) \end{array}\right].$$

The combination of Equations (36), (37), and (38) yields the following nonzero permittivity components: $\varepsilon_{22}=\varepsilon_{\|}{\cos\;}^{2}\left(\theta \right)+\varepsilon_{⊥}{\sin\;}^{2}\left(\theta \right)$, $\varepsilon_{33}=\varepsilon_{\|}{\sin\;}^{2}\left(\theta \right)+\varepsilon_{⊥}{\cos\;}^{2}\left(\theta \right)$, and $\varepsilon_{23}=\varepsilon_{32}=\left(\varepsilon_{\|}-\varepsilon_{⊥}\right)\sin\left(\theta \right)\cos\left(\theta \right)$. As in the case of the OA in the *xy* and *xz* planes, the off-diagonal elements $\varepsilon_{23}=\varepsilon_{32}$ vanish for θ=0,π/2. Given that $\varepsilon_{12}=\varepsilon_{13}=\varepsilon_{21}=\varepsilon_{31}=0$, the condition |M|=0 of the *M* matrix in Equation (7) yields

(Equation 39) $$\left(p^{2}-p_{22}^{2}\right)\left(p^{2}-\frac{\varepsilon_{11}}{\varepsilon_{33}}p_{33}^{2}\right)-\left(p^{2}+\varepsilon_{11}k_{0}^{2}\right)\frac{\varepsilon_{23}\varepsilon_{32}}{\varepsilon_{33}}k_{0}^{2}=0.$$

For $\varepsilon_{23}\varepsilon_{32}=0$, the four values of the transverse wavevector are therefore given by the corresponding ones to TE $\left(p=\pm p_{22}\right)$ and TM $\left(p=\pm \sqrt{\varepsilon_{11}/\varepsilon_{33}}p_{33}\right)$ waves, as detailed in subsection ε12ε21 = 0. For *ε*_23_*ε*_32_≠0, on the other hand, the solutions of Equation (39) are

(Equation 40a) $$p=\pm \alpha_{+},\pm \alpha_{-},$$

(Equation 40b) $$\alpha_{\pm }=\sqrt{\chi \pm \sqrt{\chi^{2}-q}},$$

(Equation 40c) $$2\chi =p_{22}^{2}+\frac{1}{\varepsilon_{33}}\left(\varepsilon_{11}p_{33}^{2}+\varepsilon_{23}\varepsilon_{32}k_{0}^{2}\right),$$

(Equation 40d) $$q=\frac{\varepsilon_{11}}{\varepsilon_{33}}\left(p_{22}^{2}p_{33}^{2}-\varepsilon_{23}\varepsilon_{32}k_{0}^{4}\right).$$

Equations (40a) and (40d) hold for any angle θ∈[0,π/2], as the values of *p* reduces to those of TE and TM waves for *ε*_23_*ε*_32_→0, as established by Equation (39). After solving Equation (7) for the electrical field components related to the four roots of *p* in Equation (40a) and applying the boundary conditions in Equations (9a) and (9b) for the relevant tangential field components, as we did it in subsection ε12ε21 = 0, one obtains the following dispersion relations

(Equation 41a) $$\begin{array}{l} \left(\frac{\alpha_{+}^{2}-p_{22}^{2}}{\alpha_{+}^{2}+\varepsilon_{11}k_{0}^{2}}\right)\left(\frac{\varepsilon_{s}\alpha_{+}+\varepsilon_{11}p_{s}\text{tanh}(\alpha_{+}d/2)}{\alpha_{+}+p_{s}\text{tanh}(\alpha_{+}d/2)}\right)=\\ \left(\frac{\alpha_{-}^{2}-p_{22}^{2}}{\alpha_{-}^{2}+\varepsilon_{11}k_{0}^{2}}\right)\left(\frac{\varepsilon_{s}\alpha_{-}+\varepsilon_{11}p_{s}\text{tanh}(\alpha_{-}d/2)}{\alpha_{-}+p_{s}\text{tanh}(\alpha_{-}d/2)}\right), \end{array}$$

(Equation 41b) $$\begin{array}{l} \left(\frac{\alpha_{+}^{2}-p_{22}^{2}}{\alpha_{+}^{2}+\varepsilon_{11}k_{0}^{2}}\right)\left(\frac{\varepsilon_{11}p_{s}+\varepsilon_{s}\alpha_{+}\text{tanh}(\alpha_{+}d/2)}{p_{s}+\alpha_{+}\text{tanh}(\alpha_{+}d/2)}\right)=\\ \left(\frac{\alpha_{-}^{2}-p_{22}^{2}}{\alpha_{-}^{2}+\varepsilon_{11}k_{0}^{2}}\right)\left(\frac{\varepsilon_{11}p_{s}+\varepsilon_{s}\alpha_{-}\text{tanh}(\alpha_{-}d/2)}{p_{s}+\alpha_{-}\text{tanh}(\alpha_{-}d/2)}\right). \end{array}$$

As in previous cases, for a thin nanofilm $\left(\lambda =k_{0}d/2<1\right)$ of interest in this work, the solutions of Equations (41a) and (41b) for the SPhP wavevector *β* can analytically be obtained through the perturbation method. For an approximation up to *λ*^2^, this method determines that the effective permittivity $\varepsilon ={\left(\beta /k_{0}\right)}^{2}$ of the SPhP mode in Equation (41a) depends on the film permittivity through its *ε*_11_ component only and is given by

(Equation 42) $$\varepsilon =\varepsilon_{s}+{\left(\frac{-\varepsilon_{s}}{\varepsilon_{11}\lambda }\right)}^{2}.$$

The transverse wavevector outside of the film $p_{s}=k_{0}\sqrt{\varepsilon -\varepsilon_{s}}=-k_{0}\varepsilon_{s}/\varepsilon_{11}\lambda$ hence establishes that the surface confinement of this SPhP mode strengthens $\left(\text{Re}\left(p_{s}\right)>0\right)$ for thinner films, provided that $\text{Re}\left(\varepsilon_{11}\right)<0$. In addition, the real and imaginary parts of the SPhP wavevector $\beta =k_{0}\sqrt{\varepsilon }=-k_{0}\left(\varepsilon_{s}/\varepsilon_{11}\lambda +\varepsilon_{11}\lambda /2\right)$ take higher values for thinner films, such that its ratio $\text{Re}\left(\beta \right)/\text{Im}\left(\beta \right)\approx -\text{Re}\left(\varepsilon_{11}\right)/\text{Im}\left(\varepsilon_{11}\right)$ becomes pretty much independent of the film thickness. This SPhP mode is thus characterized by a short propagation length ${\Lambda =\left(2\text{Im}\left(\beta \right)\right)}^{-1}∼\lambda$ and appears within a relatively narrow interval of frequencies fulfilling the condition $\text{Re}\left(\varepsilon_{11}\right)<0$. Therefore, the contribution of the SPhP mode in Equation (41a) to the thermal conductivity in Equation (1) is expected to be limited if not negligible. The dispersion relation in Equation (41b), on the other hand, has the following perturbation solutions for an approximation up to *λ*^2^ on the effective permittivity

(Equation 43a) $$\varepsilon =\varepsilon_{s}+\frac{1}{2}\left(a^{2}-2b\pm a\sqrt{a^{2}-4b}\right)\lambda^{2},$$

(Equation 43b) $$a\varepsilon_{33}=\varepsilon_{s}^{2}-\varepsilon_{22}\varepsilon_{33}+\varepsilon_{23}\varepsilon_{32},$$

(Equation 43c) $$b\varepsilon_{33}=\varepsilon_{s}\left[\left(\varepsilon_{s}-\varepsilon_{22}\right)\left(\varepsilon_{s}-\varepsilon_{33}\right)-\varepsilon_{23}\varepsilon_{32}\right].$$

For $\varepsilon_{23}\varepsilon_{32}\neq 0$ (0<θ<π/2), these two asymptotic solutions for ε are different than the typical ones obtained for the TE and TM waves found when the OA is in the *xy* and *xz* planes. This fact indicates that the propagation of SPhPs along a nanofilm with its OA in the yz plane cannot be described in terms of TE and TM waves. However, when $\varepsilon_{23}\varepsilon_{32}=0$ (θ=0,π/2), Equation (43a) reduces to $\varepsilon =\varepsilon_{s}+{\left(\varepsilon_{22}-\varepsilon_{s}\right)}^{2}\lambda^{2}$ and $\varepsilon =\varepsilon_{s}+\varepsilon_{s}^{2}{\left(1-\varepsilon_{s}/\varepsilon_{33}\right)}^{2}\lambda^{2}$, which correspond to the effective permittivities for the TE (Equation (21)) and TM (Equation (27)) waves, respectively. The off-diagonal components of the film permittivity tensor are thus responsible for the propagation of SPhPs via non-TE and non-TM waves, whose propagation wavevector $\beta =k_{0}\sqrt{\varepsilon }$ is driven by *ε*_22_, *ε*_33_, and *ε*_23_, *ε*_32_.

## Results and discussion

The propagation and thermal conductivity of the SPhPs along an hBN nanofilm is quantified and analyzed in this section. The hBN is a uniaxial anisotropic material able to support the propagation of SPhPs in a wide frequency range (Caldwell et al., 2014; Tao et al., 2021) and therefore can be considered as a good SPhP conductor. The permittivity components parallel $\left(\varepsilon_{\|}\right)$ and perpendicular $\left(\varepsilon_{⊥}\right)$ to the OA of hBN are well described by the Lorentz model (Tao et al., 2021)

(Equation 44) $$\varepsilon_{n}=\varepsilon_{\infty ,n}\left(1+\frac{\omega_{L,n}^{2}-\omega_{T,n}^{2}}{\omega_{T,n}^{2}-\omega^{2}-i\gamma_{n}\omega }\right),$$

where *ω*_*L*,*n*_ and *ω*_*T*,*n*_ are the respective longitudinal and transverse optical phonon frequencies, $\varepsilon_{\infty ,n}$ is a high-frequency permittivity constant, and *γ*_*n*_ is the damping parameter along the direction n=‖,⊥. The values of these parameters are summarized in Table 1. The permittivity components of hBN do not change significantly with temperature, for temperatures between 300 K and 700 K (Zhang et al., 2012) and therefore the calculations in this work are done with its room temperature values in Equation (44).

**Table 1:** Parameters determining the hBN permittivity components in Equation (10) (Caldwell et al., 2014; Wu and Fu 2021)

	n=⊥	n=‖
$\varepsilon_{\infty ,n}$	4.87	2.95
*ω*_*L*_,_*n*_ (Trad/s)	303	156
*ω*_*T*_,_*n*__*(Trad/s)*_	258	147
*γ*_*n*__*(Trad/s)*_	0.94	0.75

**Figure 2:**
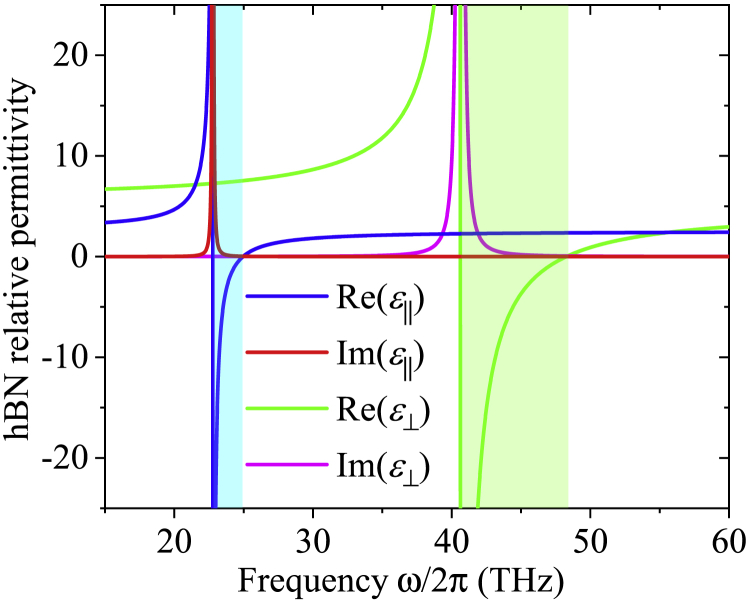
Real and imaginary parts of the hBN relative permittivity components parallel $\left(\varepsilon_{\|}\right)$ and perpendicular $\left(\varepsilon_{⊥}\right)$ to the OA The colored zones stand for the Reststrahlen bands (Re(ε)<0).

The frequency spectrum of the real and imaginary parts of the hBN relative permittivity components parallel $\left(\varepsilon_{\|}\right)$ and perpendicular $\left(\varepsilon_{\|}\right)$ to the OA is shown in Figure 2. The resonance peaks of $\text{Im}\left(\varepsilon_{\|}\right)$ and $\text{Im}\left(\varepsilon_{⊥}\right)$ at 23.4 and 41.0 THz indicate that hBN absorbs a significant amount of energy from the SPhP electromagnetic field and therefore limits its propagation at those frequencies. By contrast, the dips of $\text{Re}\left(\varepsilon_{\|}\right)$ and $\text{Re}\left(\varepsilon_{⊥}\right)$ occur at 23.5 and 41.1 THz that represent the frequencies at which the SPhPs usually exhibit the strongest confinement to the interface (Ordonez-Miranda et al., 2021; Wu et al., 2020). The colored zones, on the other hand, stand for the Reststrahlen bands $\left(\text{Re}\left(\varepsilon_{n}\right)<0\right)$ defining the range of frequencies that would support the propagation of SPhPs via TM waves in absence of absorption $\left(\text{Im}\left(\varepsilon_{n}\right)=0\right)$ (Ordonez-Miranda et al., 2021; Wu et al., 2020). However, given that hBN is an absorbing material $\left(\text{Im}\left(\varepsilon_{n}\right)>0\right)$, SPhPs are expected to propagate with frequencies inside and outside of these bands, as reported for isotropic nanofilms (Ordonez-Miranda et al., 2021) and is shown below.

According to the results obtained in subsection ε12ε21 = 0 for the OA in the xy axis, the strong coupling propagation of SPhPs along the upper and lower interfaces of a nanofilm (λ≪1) is described by even (TM-like) and odd (TE-like) modes appearing for an arbitrary angle *θ*. The SPhP confinement condition $\left(\text{Re}\left(p_{s}\right)>0\right)$ along with Equations (21) and (27) establishes that these modes appear in the range of frequencies determined by the inequalities *J*_*n*_>0, where

(Equation 45a) $$J_{even}=1-\frac{\varepsilon_{s}\text{Re}\left(\varepsilon_{33}\right)}{{\left|\varepsilon_{33}\right|}^{2}},$$

(Equation 45b) $$J_{odd}=\text{Re}\left(\varepsilon_{22}\right)-\varepsilon_{s}.$$

**Figure 3:**
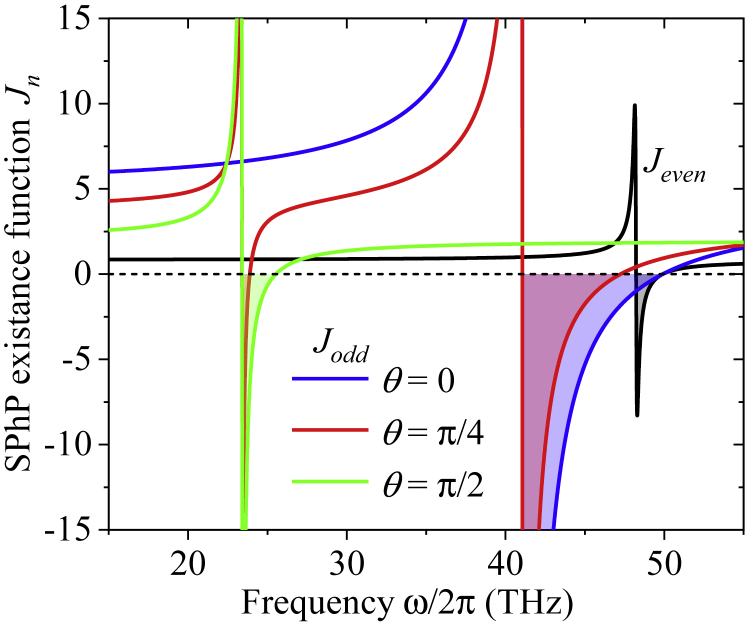
Frequency spectrum of the SPhP existence functions *J*_*even*_ and *J*_*odd*_ of an hBN nanofilm with its OA in the xy plane and surrounded by vacuum $\left(\varepsilon_{s}=1\right)$ The colored areas represent the band gaps for the existence and propagation of SPhPs.

The existence of SPhPs along a thin nanofilm (λ≪1) is thus determined by its permittivity components perpendicular to their propagation direction (x axis) and the permittivity of its surrounding medium. While the odd mode exists for all frequencies fulfilling the condition $\text{Re}\left(\varepsilon_{22}\right)>\varepsilon_{s}\left(=1\text{ for vacuum or air}\right)$, the even one appears for positive and negative values of $\text{Re}\left(\varepsilon_{33}\right)$. In this latter case, Equation (45a) shows that the range of allowed frequencies reduces to those within the negative peak of $\text{Re}\left(\varepsilon_{33}\right)<0$ (Reststrahlen band) for $\varepsilon_{s}\to \infty$ only. For any other case of practical interest, the interval of frequencies supporting the propagation of SPhPs is usually much broader than the Reststrahlen band, as was reported in the literature (Chen and Chen 2007; Gluchko et al., 2017; Ordonez-Miranda et al., 2021) for isotropic films and is predicted by the existence function *J*_*even*_ shown in Figure 3. When the OA is in the xy plane, $\varepsilon_{33}=\varepsilon_{⊥}$ (see Equation (11)) and therefore the frequency band gap of *J*_*even*_ (gray zone) is independent of the angle *θ* and shows up just above the corresponding Reststrahlen band (greenish zone in Figure 2). By contrast, the band gaps (colored areas) of *J*_*add*_ in Figure 3 do involve frequencies inside the corresponding Reststrahlen bands of $\varepsilon_{⊥}$ and $\varepsilon_{\|}$ and depend on *θ* Z*θ* due to the function *ε*_22_(*θ*) defined just below Equation (12). As SPhPs do not exist within these frequency band gaps, they have to be excluded from the integral in Equation (1).

**Figure 4:**
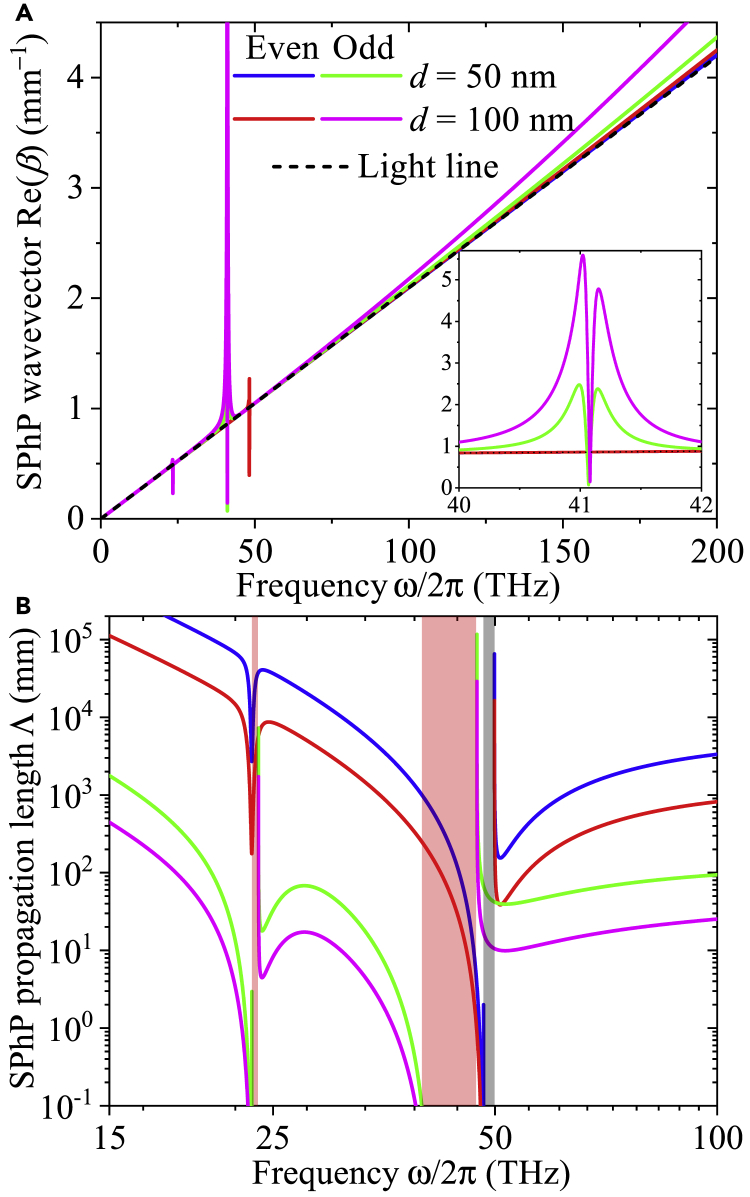
Propagation parameters of SPhPs SPhP in-plane (a) wavevector Re(I) and (b) propagation length $\Lambda ={\left[2\text{Im}\left(\beta \right)\right]}^{-1}$ as functions of frequency (Ordonez-Miranda et al., 2013), for the even and odd modes propagating along an hBN nanofilm with its OA in the *xy* plane and surrounded by vacuum (*ε*_*s*_ = 1). The legend is (a) also holds for (b), where the colored regions represent the range of frequencies for which there is no propagation of SPhPs. Calculations were done for *θ* = π/4.

The frequency dependence of the in-plane wavevector Re(*β*) and propagation length Λ of the even and odd modes of SPhPs propagating along the interfaces of a 50-nm-thick hBN nanofilm suspended in vacuum (or air) are shown in Figures 4A and 4B, respectively. For both modes, Re(*β*) generally increases with the film thickness and frequency through values very close to those of the light line (*k*_0_ = *ω*/*c*). The SPhPs thus show a photon-like behavior characterized by a group velocity V=∂ω/∂Re(β) close to that of light in vacuum (*V*→*c*). As the frequency increases, *V* decreases due to the increase of Re(*β*) through values higher than those of the light line. The fact that Re(*β*)(odd mode)>Re(*β*)(even mode) indicates that the SPhP odd mode is slower than the even one. In addition, for a given frequency, the propagation length (>0.1 mm) of the even mode is much longer than the corresponding one of the odd mode, as shown in Figure 4B. Note that both modes exhibit frequency band gaps (colored regions) for which there is no propagation of SPhPs, in agreement with the predictions of the SPhP existence functions shown in Figure 3. As the band gap of the even mode is narrower than the thicker one of the odd mode, the former mode supports the propagation of SPhPs in a wider range of frequencies than the latter one. Based on these facts, it is clear than the trade-off between Re(*β*) and Λ is better optimized by the even mode via the product Re(β)Λ, which enhances the SPhP thermal conductivity defined in Equation (1). Furthermore, as the increase of Re(*β*) with the film thickness (Figure 4A) is relatively small with respect to the corresponding reduction of Λ for both modes (Figure 4B), the product Re(β)Λ and hence the SPhP thermal conductivity (Equation (1)) is expected to take lower values of thicker films.

**Figure 5:**
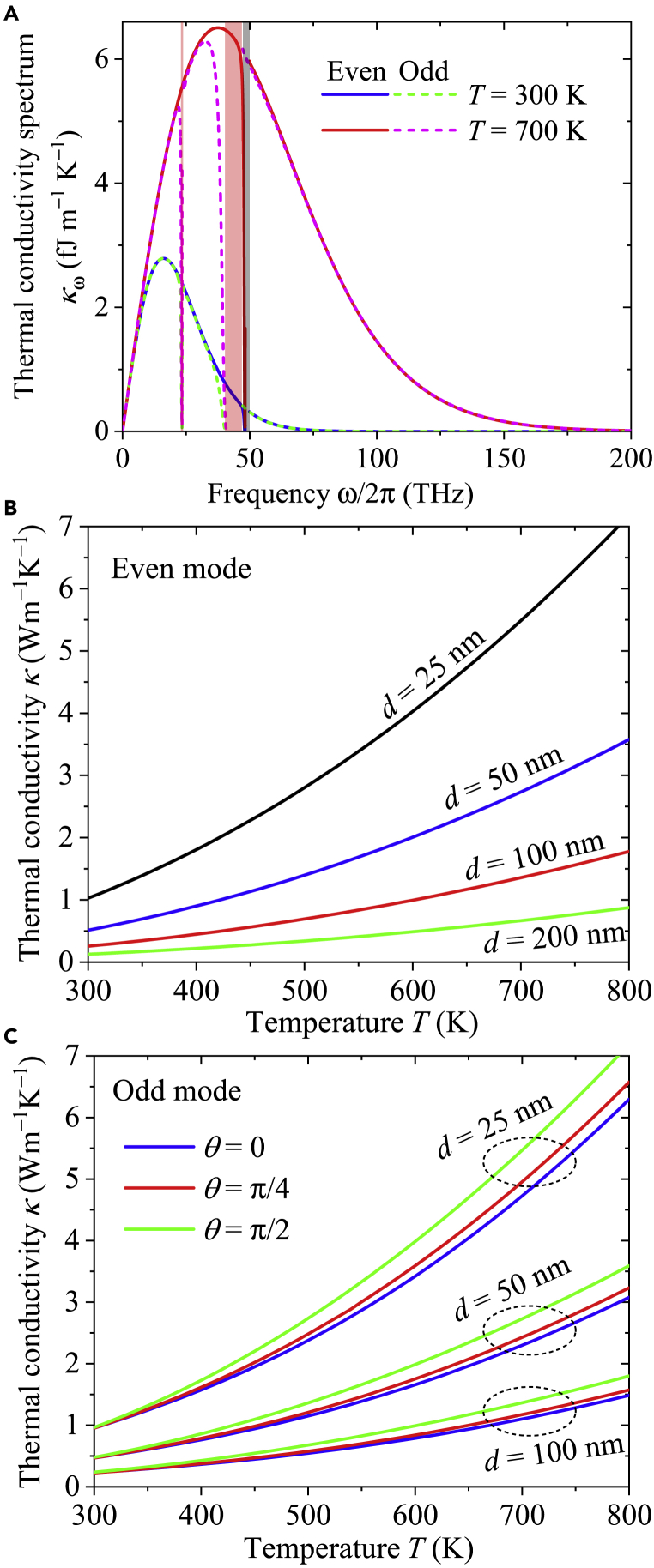
SPhP thermal conductivity of an hBN nanofilm SPhP thermal conductivity spectrum along with its integrated values for the (b) even and (c) odd modes as functions of the average temperature of an hBN nanofilm with its OA in the *xy* plane and surrounded by vacuum (*ε*_*s*_ = 1). Calculations were done for *d* = 50 nm, *θ* = *π*/4, *l* = 1 mm, and two representative temperatures.

Figure 5A shows the SPhP thermal conductivity spectrum $\kappa_{\omega }$ established by Equation (1) $\left(\kappa =\int \kappa_{\omega }d\omega \right)$ and calculated with the results shown in Figure 4 for the even and odd modes of SPhPs propagating along an hBN nanofilm. Even though the spectra of both SPhP modes are pretty much the same for two representative temperatures, the contribution of the even mode is expected to be higher than that of the odd one, due to its relatively narrow frequency band gap. The negligible difference between the values of $\kappa_{\omega }$ for the even and odd modes, and any of their allowed frequencies, arises from the fact that the effective propagation length for both modes is nearly equal to the nanofilm length $\left(\Lambda_{e}\approx l\right)$, due to the SPhP ballistic propagation (Λ≫l) for the considered length *l* = 1 mm that is typically used in practice (Wu et al., 2020), for a suspended nanofilm. Note that the major contribution to $\kappa_{\omega }$ arises from frequencies ω/2*π<*100 THz in which the SPhPs propagate with pretty much the same wavevector (and speed) than light, as shown in Figure 4A. The photon-like nature of SPhPs is thus responsible of their main contribution to the SPhP thermal conductivity. Given that in the ballistic regime the product $\text{Re}\left(\beta \right)\Lambda_{e}\approx k_{0}l$, Equation (1) establishes that the maximum contribution to *κ*_*ω*_ shows up at the characteristic frequency $\omega_{c}/2\pi$ (THz) =0.05367T (K), which represents the Wien’s displacement law for ballistic SPhPs. This linear relation of *ω*_*c*_ with *T* drives the increase of the SPhP thermal conductivity *κ* with temperature, as shown in Figures 5B and 5C. While the contribution of the even mode is independent of the angle *θ*, as predicted by Equation (27) for $\varepsilon_{33}=\varepsilon_{⊥}$, the one of the odd mode exhibits a slight increase with *θ*, specially at high temperature. For a given temperature, the minimum thermal conductivity is obtained with the OA aligned (*θ* = 0) with the SPhP propagation direction (*x* axis), while its maximum is achieved when the OA is along the *y* axis (*θ* = *π*/2) and therefore perpendicular to the SPhP direction. The increase of κ(odd mode) with *θ* is, however, less significant than those observed with the rising of temperature and reduction of the film thickness. This behavior of κ(odd mode) and κ(even mode) with *T* and *d* is opposite to the typical one exhibited by the phonon thermal conductivity and therefore it represents the fingerprint of the SPhP heat transport, as reported in the literature (Chen et al., 2005; Ordonez-Miranda et al., 2013; Tranchant et al., 2019; Wu et al., 2020). Furthermore, note that for any *T* and *d*, the contributions of the even and odd modes are quite similar and therefore both have a sizable contribution to the total (κ(even mode)+κ(odd mode)) SPhP thermal conductivity. Very similar results for the propagation parameters and SPhP thermal conductivity are obtained for an hBN nanofilm with its axis in the *xz* and *yz* planes, as shown in the supplementary material. This fact numerically confirms that the SPhP heat transport along an anisotropic hBN nanofilm is pretty much independent of the orientation of its OA and therefore has a nearly isotropic behavior.

The near-isotropic SPhP thermal conductivity values obtained for an hBN nanofilm thicker than 25 nm are much smaller than the typical ones (from 200 to 300 Wm^−1^K^−1^) of the phonon counterpart for a suspended nanofilm with a thickness of a few nanometers and temperatures higher than room temperature (Jo et al., 2013). However, since the SPhP thermal conductivity is proportional to *d*^−3^, as established by Equation (1) for the ballistic regime, the SPhP heat transport is expected to be comparable to that of phonons in a crystalline hBN film with a thickness *d* of a few nanometers. On the other hand, in amorphous hBN with a relatively low phonon thermal conductivity (around 3 m^−1^K^−1^), the SPhP thermal conductivity could even become the dominant contribution as the film thickness reduces to nanoscales.

### Conclusions

We have theoretically shown that the heat transport of surface phonon-polaritons propagating along a uniaxial anisotropic nanofilm is nearly isotropic despite of the strong anisotropy of its permittivity components. This has been done by deriving simple and analytical expressions for the polariton in-plane wavevector, whose real and imaginary parts drives the polariton thermal conductivity of a polar nanofilm. It has been shown that the propagation of polaritons is determined by even and odd modes that generalize the transverse magnetic and transverse electrical ones that typically appear in isotropic films. The frequency spectrum of these generalized modes can efficiently be tuned with the angle between the nanofilm optical axis and their propagation direction. For an hBN nanofilm, both the even and odd modes have a remarkable contribution to the total polariton thermal conductivity, which takes a value higher than 5.6 Wm^−1^K^−1^ for a 25-nm-thick nanofilm at 500 K, when the optical axis is in its plane. Even though the polariton thermal conductivity increases for a thinner and/or hotter nanofilm, its dependence on the orientation of its optical axis is weak, even at high temperature. This near-isotropic response of an hBN nanofilm results from the polariton ballistic behavior induced by the ultra-long propagation lengths (>1 mm) for most frequencies. The obtained results thus highlight key features of the propagation and heat transport of anisotropy-driven polaritons and could be useful for improving the heat dissipation along polar nanostructures.

### Limitations of the study

As pointed out in section Theoretical modeling, the obtained analytical expressions for the effective permittivity are valid for nanofilms typically thinner than 200 nm. For these thicknesses, the temperature gradient across the nanofilm can be neglected and Equation (1) accurately predicts its SPhP thermal conductivity.

## STAR★Methods

### Key resources table

**Table:** 

REAGENT or RESOURCE	SOURCE	IDENTIFIER
**Deposited data**		

Propagation parameters	www.joseordonez.cnrs.fr	Figures 2, 3 and 4
Thermal conductivity	www.joseordonez.cnrs.fr	Figure 5

**Software and algorithms**		

Mathematica	www.wolfram.com	Mathematica 13.0

### Resource availability

#### Lead contact

Further information and requests for resources including data and code should be directed to and will befulfilled by the lead contact, Jose Ordonez-Miranda (jose.ordonez@cnrs.fr).

#### Materials availability

This study did not generate new unique reagents.

#### Method details

All equations were derived from the Maxwell equations of electromagnetism and their predictions were analyzed through the software Mathematica 13.0.

## Data Availability

• There are two datasets: one for the propagation parameters and one for the thermal conductivity. Accession numbers are listed in the key resources table. • All algorithms are implemented in the computational software Wolfram Mathematica. All original codes are deposited and publicly available on www.joseordonez.cnrs.fr. Instructions how to run this code. • Any additional information required to reanalyze the data reported in this paper is available from the lead contact upon request.

## References

[bib1] Agranovich V.M. (2012).

[bib2] Alvarez-Perez G., Voronin K.V., Volkov V.S., Alonso-González P., Nikitin A.Y. (2019). Analytical approximations for the dispersion of electromagnetic modes in slabs of biaxial crystals. Phys. Rev. B.

[bib3] Barra-Burillo M., Muniain U., Catalano S., Autore M., Casanova F., Hueso L.E., Aizpurua J., Esteban R., Hillenbrand R. (2021). Microcavity phonon polaritons from the weak to the ultrastrong phonon–photon coupling regime. Nat. Commun..

[bib4] Caldwell J.D., Kretinin A.V., Chen Y., Giannini V., Fogler M.M., Francescato Y., Ellis C.T., Tischler J.G., Woods C.R., Giles A.J. (2014). Sub-diffractional volume-confined polaritons in the natural hyperbolic material hexagonal boron nitride. Nat. Commun..

[bib5] Chen D.Z.A., Chen G. (2007). Measurement of silicon dioxide surface phonon-polariton propagation length by attenuated total reflection. Appl. Phys. Lett..

[bib6] Chen D.-Z.A., Narayanaswamy A., Chen G. (2005). Surface phonon-polariton mediated thermal conductivity enhancement of amorphous thin films. Phys. Rev. B.

[bib7] Dai S., Fei Z., Ma Q., Rodin A.S., Wagner M., McLeod A.S., Liu M.K., Gannett W., Regan W., Watanabe K. (2014). Tunable phonon polaritons in atomically thin van der waals crystals of boron nitride. Science.

[bib8] Gluchko S., Palpant B., Volz S., Braive R., Antoni T. (2017). Thermal excitation of broadband and long-range surface waves on sio2 submicron films. Appl. Phys. Lett..

[bib9] Greffet J.-J., Carminati R., Joulain K., Mulet J.-P., Mainguy S., Chen Y. (2002). Coherent emission of light by thermal sources. Nature.

[bib10] Guo Y., Tachikawa S., Volz S., Nomura M., Ordonez-Miranda J. (2021). Quantum of thermal conductance of nanofilms due to surface-phonon polaritons. Phys. Rev. B.

[bib11] Jacob Z., Narimanov E.E. (2008). Optical hyperspace for plasmons: dyakonov states in metamaterials. Appl. Phys. Lett..

[bib12] Jo I., Pettes M.T., Kim J., Watanabe K., Taniguchi T., Yao Z., Shi L. (2013). Thermal conductivity and phonon transport in suspended few-layer hexagonal boron nitride. Nano Lett..

[bib13] Li R., Cheng C., Ren F.-F., Chen J., Fan Y.-X., Ding J., Wang H.-T. (2008). Hybridized surface plasmon polaritons at an interface between a metal and a uniaxial crystal. Appl. Phys. Lett..

[bib14] Liu R., Ge L., Wu B., Cui Z., Wu X. (2021). Near-field radiative heat transfer between topological insulators via surface plasmon polaritons. iScience.

[bib15] Luo R., Gu Y., Li X., Wang L., Khoo I.-C., Gong Q. (2013). Mode recombination and alternation of surface plasmons in anisotropic mediums. Appl. Phys. Lett..

[bib16] Ma W., Alonso-González P., Li S., Nikitin A.Y., Yuan J., Martín-Sánchez J., Taboada-Gutiérrez J., Amenabar I., Li P., Vélez S. (2018). In-plane anisotropic and ultra-low-loss polaritons in a natural van der waals crystal. Nature.

[bib17] Ma W., Hu G., Hu D., Chen R., Sun T., Zhang X., Dai Q., Zeng Y., Alu A., Qiu C.-W., Li P. (2021). Ghost hyperbolic surface polaritons in bulk anisotropic crystals. Nature.

[bib18] Maia F.C.B., O’Callahan B.T., Cadore A.R., Barcelos I.D., Campos L.C., Watanabe K., Taniguchi T., Deneke C., Belyanin A., Raschke M.B., Freitas R.O. (2019). Anisotropic flow control and gate modulation of hybrid phonon-polaritons. Nano Lett..

[bib19] Ordonez-Miranda J., Tranchant L., Tokunaga T., Kim B., Palpant B., Chalopin Y., Antoni T., Volz S. (2013). Anomalous thermal conductivity by surface phonon-polaritons of polar nano thin films due to their asymmetric surrounding media. J. Appl. Phys..

[bib20] Ordonez-Miranda J., Tranchant L., Chalopin Y., Antoni T., Volz S. (2014). Thermal conductivity of nano-layered systems due to surface phonon-polaritons. J. Appl. Phys..

[bib21] Ordonez-Miranda J., Tranchant L., Kim B., Chalopin Y., Antoni T., Volz S. (2014). Effects of anisotropy and size of polar nano thin films on their thermal conductivity due to surface phonon-polaritons. Appl. Phys. Express.

[bib22] Ordonez-Miranda J., Tranchant L., Kim B., Chalopin Y., Antoni T., Volz S. (2014). Quantized thermal conductance of nanowires at room temperature due to zenneck surface-phonon polaritons. Phys. Rev. Lett..

[bib23] Ordonez-Miranda J., Volz S., Nomura M. (2021). Surface phonon-polariton heat capacity of polar nanofilms. Phys. Rev. Appl..

[bib24] Segura A., Artús L., Cuscó R., Taniguchi T., Cassabois G., Gil B. (2018). Natural optical anisotropy of h-bn: highest giant birefringence in a bulk crystal through the mid-infrared to ultraviolet range. Phys. Rev. Mater..

[bib25] Tao Z.H., Dong H.M., Milošević M.V., Peeters F.M., Van Duppen B. (2021). Tailoring Dirac plasmons via anisotropic dielectric environment by design. Phys. Rev. Appl..

[bib26] Tranchant L., Hamamura S., Ordonez-Miranda J., Yabuki T., Vega-Flick A., Cervantes-Alvarez F., Alvarado-Gil J.J., Volz S., Miyazaki K. (2019). Two-dimensional phonon polariton heat transport. Nano Lett..

[bib27] Volz S., Ordonez-Miranda J., Shchepetov A., Prunnila M., Ahopelto J., Pezeril T., Vaudel G., Gusev V., Ruello P., Weig E.M. (2016). Nanophononics: state of the art and perspectives. Eur. Phys. J. B.

[bib28] Volz S., Shiomi J., Nomura M., Miyazaki K. (2016). Heat conduction in nanostructured materials. J. Therm. Sci. Technol..

[bib29] Wang X., Wang P., Chen J., Lu Y., Ming H., Zhan Q. (2011). Theoretical and experimental studies of surface plasmons excited at metal-uniaxial dielectric interface. Appl. Phys. Lett..

[bib30] Wu X., Fu C. (2021). Hyperbolic volume and surface phonon polaritons excited in an ultrathin hyperbolic slab: connection of dispersion and topology. Nanoscale Microscale Thermophys. Eng..

[bib31] Wu Y., Ordonez-Miranda J., Gluchko S., Anufriev R., Meneses D.D.S., Del Campo L., Volz S., Nomura M. (2020). Enhanced thermal conduction by surface phonon-polaritons. Sci. Adv..

[bib32] Yang F., Sambles J.R., Bradberry G.W. (1991). Long-range surface modes supported by thin films. Phys. Rev. B Condens. Matter.

[bib33] Zhang T., Wu M.-Q., Zhang S.-R., Xiong J., Wang J.-M., Zhang D.-H., He F.-M., Li Z.-P. (2012). Permittivity and its temperature dependence in hexagonal structure BN dominated by the local electric field. Chin. Phys. B.

[bib34] Zheng Z., Sun F., Huang W., Jiang J., Zhan R., Ke Y., Chen H., Deng S. (2020). Phonon polaritons in twisted double-layers of hyperbolic van der waals crystals. Nano Lett..

[bib35] Zou Y., Chakravarty S., Chung C.J., Xu X., Chen R.T. (2018). Mid-infrared silicon photonic waveguides and devices [Invited]. Photonics Res..

