# Supplementary material for: Near-isotropic polariton heat transport along a polar anisotropic nanofilm
Source: iScience. 2022 Aug 5;25(9):104857. doi: 10.1016/j.isci.2022.104857 (PMC9420522; doi:10.1016/j.isci.2022.104857)
Supplement: Document S1. Figures S1–S5 [file mmc1.pdf]

iScience, Volume *25*

Supplemental information

Near-isotropic polariton heat transport along a polar anisotropic nanofilm

Jose Ordonez-Miranda, Yunhui Wu, Masahiro Nomura, and Sebastian Volz

Supplementary Material

Near-Isotropic Polariton Heat Transport along a Polar Anisotropic Nanofilm

Jose Ordonez-Miranda, Yunhui Wu, Masahiro Nomura, Sebastian Volz

For the sake of clarity and completeness, here we show the propagation parameters and polariton thermal conductivity of a nanofilm with its optical axis in the xz and yz planes, as shown in Fig. 1 of the main manuscript.

OA in the xz plane

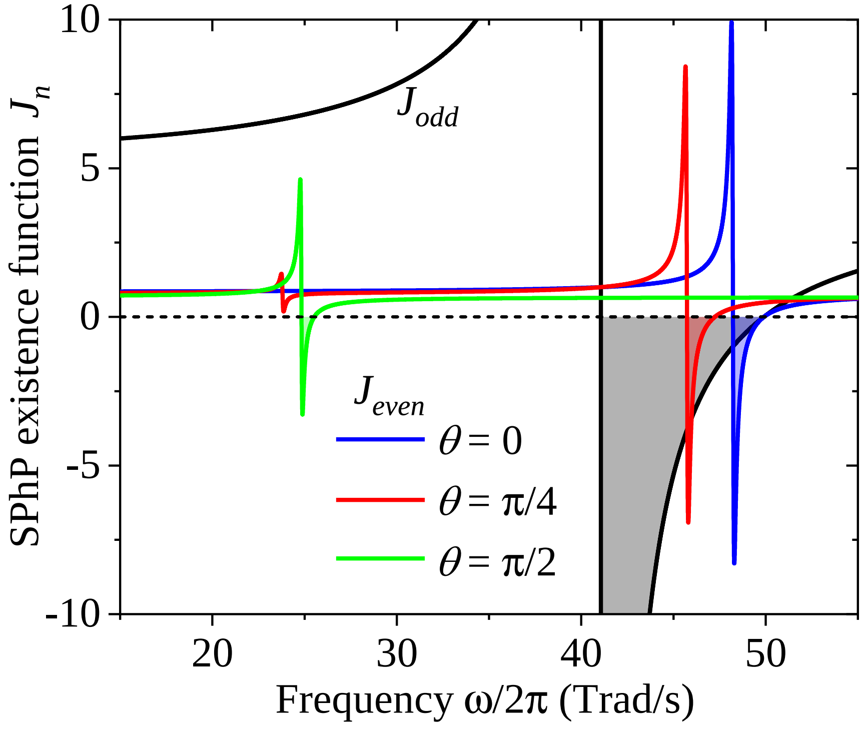

Figura S1: The same than in Fig. 3, but for the OA in the xz plane.

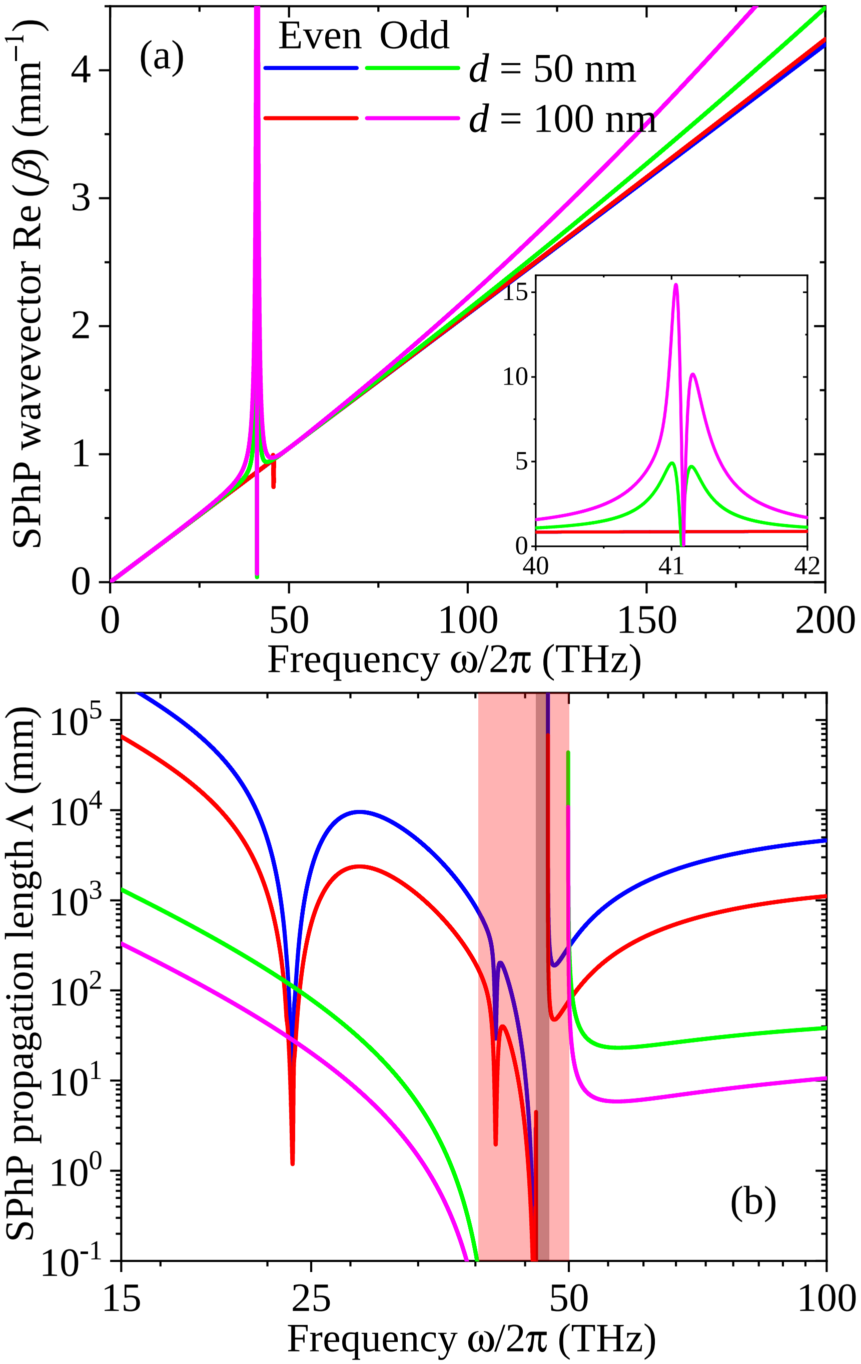

Figura S2: The same than in Fig. 4, but for the OA in the xz plane.

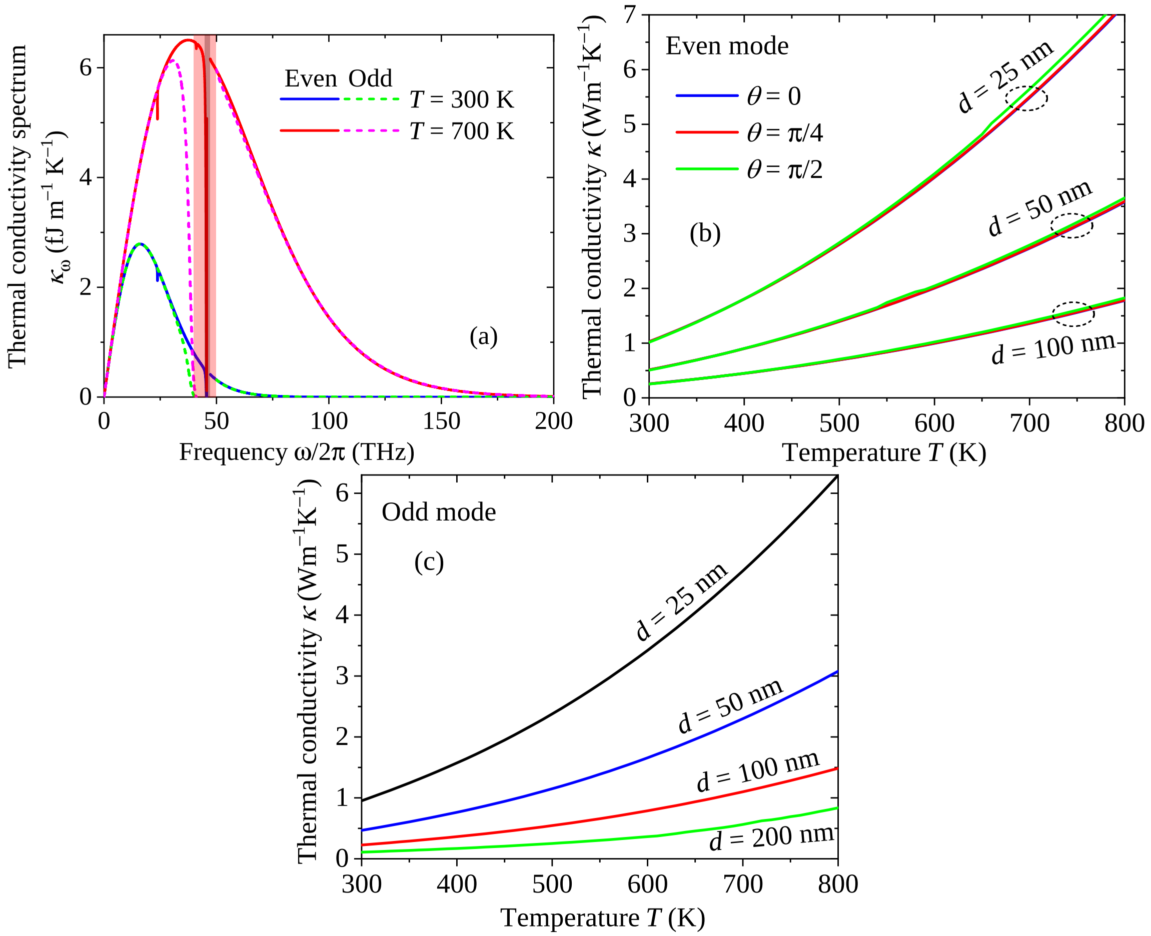

Figura S3: The same than in Fig. 5, but for the OA in the xz plane.

OA in the yz plane

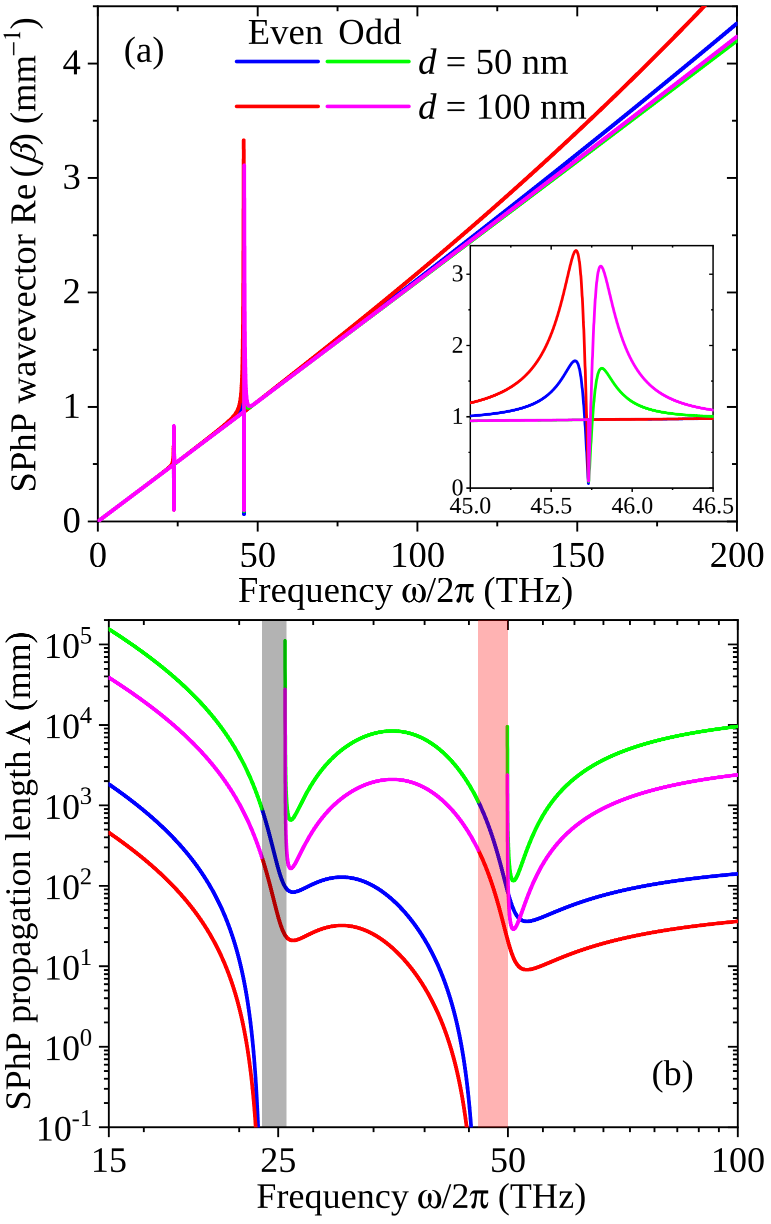

Figura S4: The same than in Fig. 4, but for the OA in the yz plane.

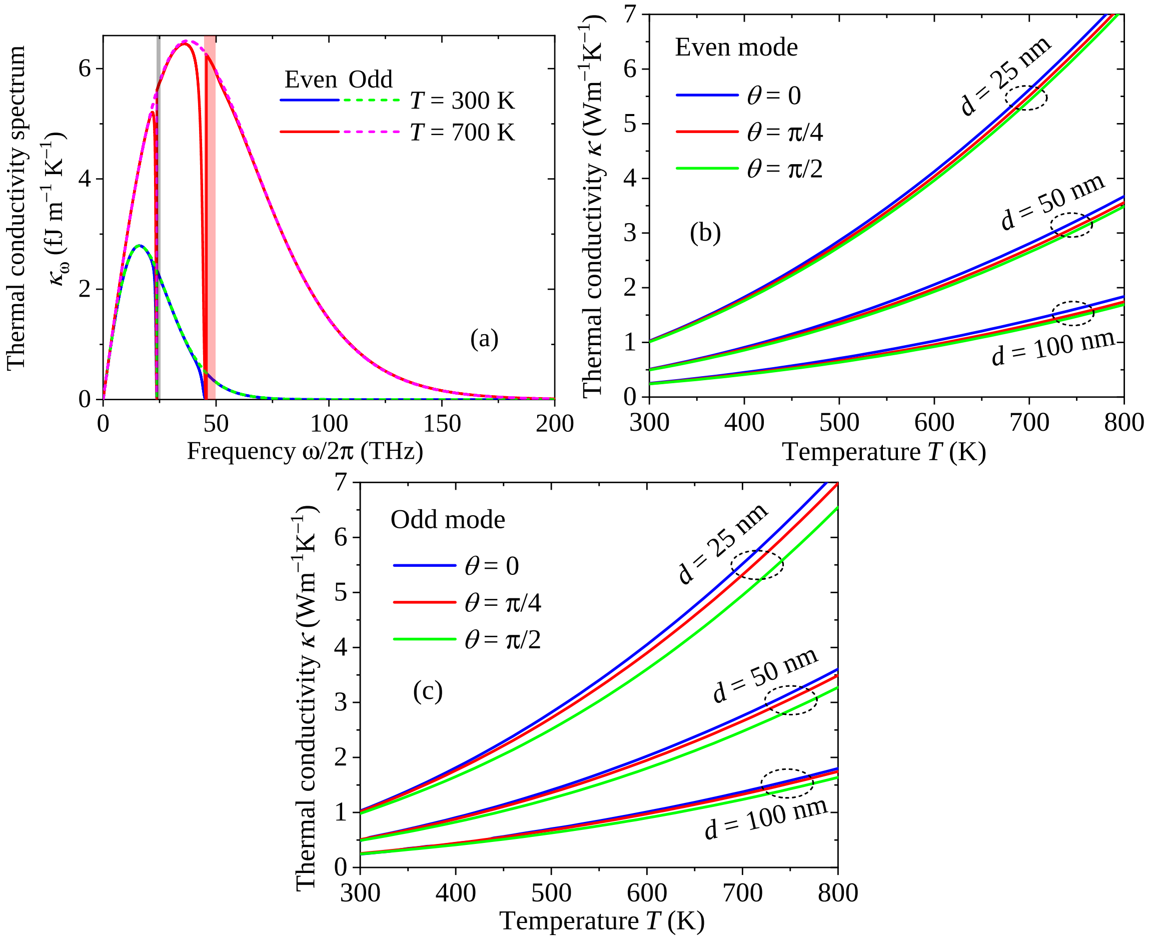

Figura S5: The same than in Fig. 5, but for the OA in the yz plane.
